# The combination of polyphenols and phospholipids as an efficient platform for delivery of natural products

**DOI:** 10.1038/s41598-023-29237-0

**Published:** 2023-02-13

**Authors:** Hassan Hashemzadeh, Mohammad Yahya Hanafi-Bojd, Milad Iranshahy, Asghar Zarban, Heidar Raissi

**Affiliations:** 1 Department of Pharmaceutics and Pharmaceutical Nanotechnology, School of Pharmacy, Birjand University of Medical Sciences, Birjand, Iran; 2 Cellular and Molecular Research Center, Birjand University of Medical Sciences, Birjand, Iran; 3 Department of Pharmacognosy, School of Pharmacy, Mashhad University of Medical Sciences, Mashhad, Iran; 4 Department of Clinical Biochemistry, Faculty of Medicine, Birjand University of Medical Sciences, Birjand, Iran; 5 Department of Chemistry, University of Birjand, Birjand, Iran; 6 Department of Chemistry & Biochemistry, Wilfrid Laurier University, Waterloo, Canada

**Keywords:** Biophysics, Computational biology and bioinformatics

## Abstract

Although nature is a rich source of potential drugs and drug leads, the widespread application of natural products (NPs) is limited due to their poor absorption when administered orally. A strategy of using phytosome has emerged as a promising technique to increase the bioavailability of NPs. Here, a comprehensive computational investigation is performed to explore the nature of interactions in the formation of phytosomes between phosphatidylcholine (PC) and a series of polyphenols (PP), including epigallocatechin-3-gallate (Eg), luteolin (Lu), quercetin (Qu), and resveratrol (Re). Our quantum mechanical calculation revealed that the intermolecular hydrogen bonds (HBs) of phosphate and glycerol parts of PC with the polyphenol compounds are the main driving force in the formation of phytosomes. The strongest HB (with energy HB = − 108.718 kJ/mol) is formed between the Eg molecule and PC. This hydrogen bond results from the flexible structure of the drug which along with several van der Waals (vdW) interactions, makes Eg-PC the most stable complex (adsorption energy = − 164.93 kJ/mol). Energy decomposition analysis confirms that the electrostatic interactions (hydrogen bond and dipole-diploe interactions) have a major contribution to the stabilization of the studied complexes. The obtained results from the molecular dynamics simulation revealed that the formation of phytosomes varies depending on the type of polyphenol. It is found that the intermolecular hydrogen bonds between PP and PC are a key factor in the behavior of the PP-PC complex in the self-aggregation of phytosome. In Eg-PC, Lu-PC, and Qu-PC systems, the formation of strong hydrogen bonds (H_BCP_ < 0 and ∇^2^ρ_BCP_ > 0) between PP and PC protects the PP-PC complexes from degradation. The steered molecular dynamics simulation results have a good agreement with experimental data and confirm that the phytosome platform facilitates the penetration of PP compounds into the membrane cells.

## Introduction

Conventional chemotherapeutic drugs suffer mainly from severe drawbacks like lack of selectivity of the tumor from normal cells, poor water solubility, and high cytotoxicity. Therefore, many researchers are trying to find novel and safe strategies to minimize the side effects of therapeutic agents^1–5^. Nature is a rich source of potential drugs and over the half of anticancer agents are inspired from natural compounds^6–8^. Natural products (NPs) can be classified into various structural classes, including alkaloids, terpenoids, organosulfur compounds, and polyphenols (PPs)^9^.

Natural PPs are bioactive natural products structurally distinguished by the presence of two or more phenol units. The existence of hydroxyl groups and conjugated bonds in benzene rings has made these compounds biologically active^9^. It is known that PP compounds could inhibit the development of tumor cells^10–14^. It must be noted that the structural differences between the different types of polyphenols significantly affect their absorption, metabolism, and their bioactivities in vivo. Nevertheless, the widespread application of these NPs is limited due to being poorly absorbed when administered orally^15,16^. The poor adsorption behavior mainly arises from two properties of polyphenols; (i) the relatively large chemical structure of polyphenols that prevent their passive diffusion, and (ii) the poor solubility of these products in lipid media that leads their crossing through the cell membrane challenging^17^.

**Figure 1:**
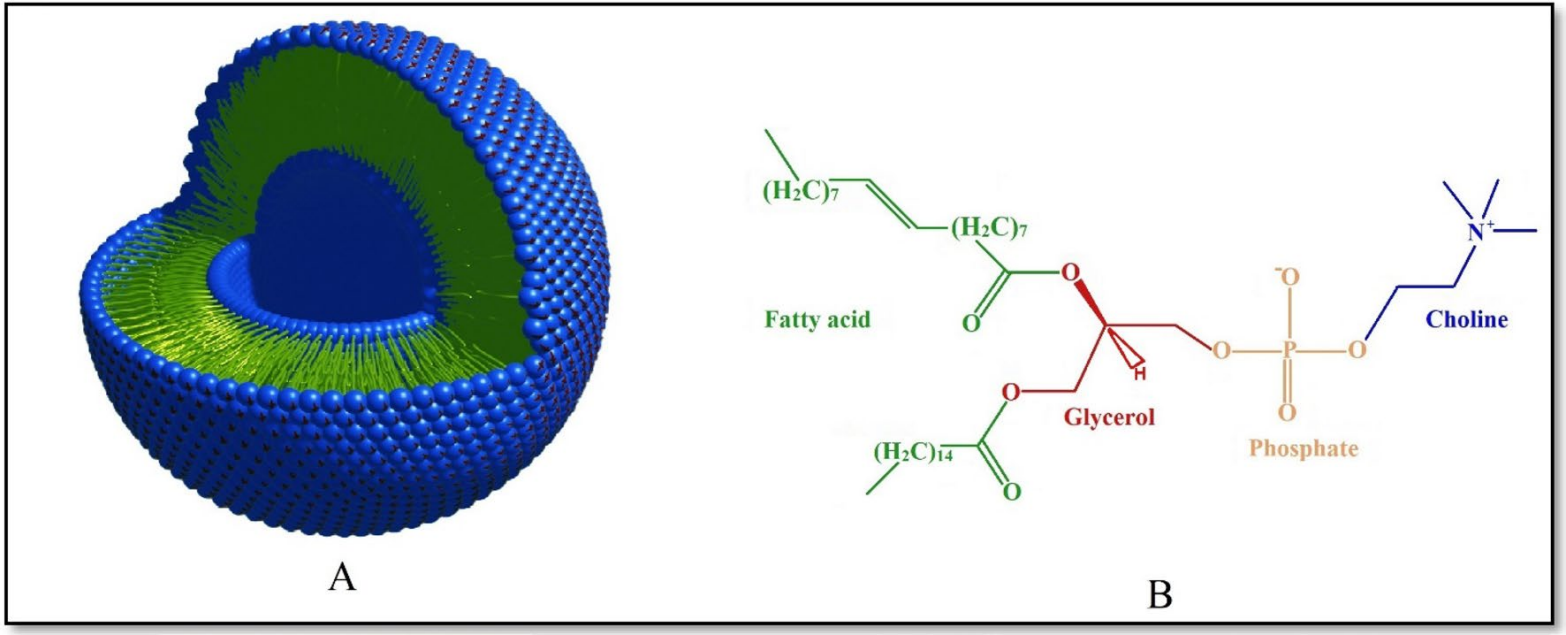
Structures of (**A**) phytosome and (**B**) phosphatidylcholine.

A variety of solutions have been proposed to increase the bio-distribution of polyphenolic agents and enhance their poor absorption^18^. Lipid-based nanocarriers, such as liposomesm^19^, nanoparticles^2,20^, prodrugs^21^, and phytosomes^22^, were reported to improve the therapeutic efficacy of natural compounds. Among the potential designed platforms, phytosome has emerged as a promising strategy to increase the bioavailability of neutral products^23–25^. Phytosomes, also called phyto-phospholipid complexes, are the vesicular complex formed by the interaction between NPs and phospholipids (Fig. 1a). The phytosome complexes are more readily absorbed and bring higher bioavailability compared to free NPs. Thanks to the special structure of phytosome, active constituents, with help of amphipathic phospholipids, easily pass through the outer membrane of gastrointestinal cells, eventually reaching the blood. It is suggested that hydrogen bonds (HBs) between the head group of phospholipid and PP are responsible for the stability of phytosome complexes^26^. However, to the best of our knowledge, no study has directly shown the role of HB in phytosome formations.

As mentioned above, the phytosome is constructed from a phospholipid part and an active agent part. Phosphatidylcholine (PC, Fig. 1b) is the most frequently employed phospholipid in making phytosomes. The chemical structure of PC is built from a phospholipid that is covalently bound to a choline group. PC is a biocompatible molecule with low toxicity, and it is one of the main components of membrane cells.

Epigallocatechin-3-gallate (EG), luteolin (Lu), quercetin (Qu), and resveratrol (Re) have belonged to the polyphenol family that can be used in treatment many diseases^27–31^. The clinical application of these compounds is limited due to low chemical stability, poor solubility, and poor bioavailability. Marwah and coworkers reported the EG upon the complex with PC, in a liposomal system, provides a stable drug delivery system with a controlled release property^32^. Luo et al. designed a drug delivery system (DDS) based on the formed complex between Re, PC, and self-micro emulsifying^33^. Their obtained results indicated that this system enhances the oral bioavailability of Re. Other similar studies have been performed on the formation of a complex between Lu and Qu drugs with PC^34,35^.

In this work, we conducted a comprehensive computational study to explore the main driving forces in the formation of phytosomes between PC and a series of polyphenols, including Eg, Lu, Qu, and Re. The nature of intermolecular interactions between PC and PPs is investigated using molecular mechanics (MM) and quantum mechanical (QM) calculations. The possibility of the phytosome formation from the self-aggregation of PC-PP molecules in an aqueous environment is evaluated by all-atoms molecular dynamics (MD) simulation. Finally, using steered MD (SMD) simulations, the diffusion of the formed phytosomes into a membrane cell is examined.

## The strategy of calculations

### Molecular models and initial structures

**Figure 2:**
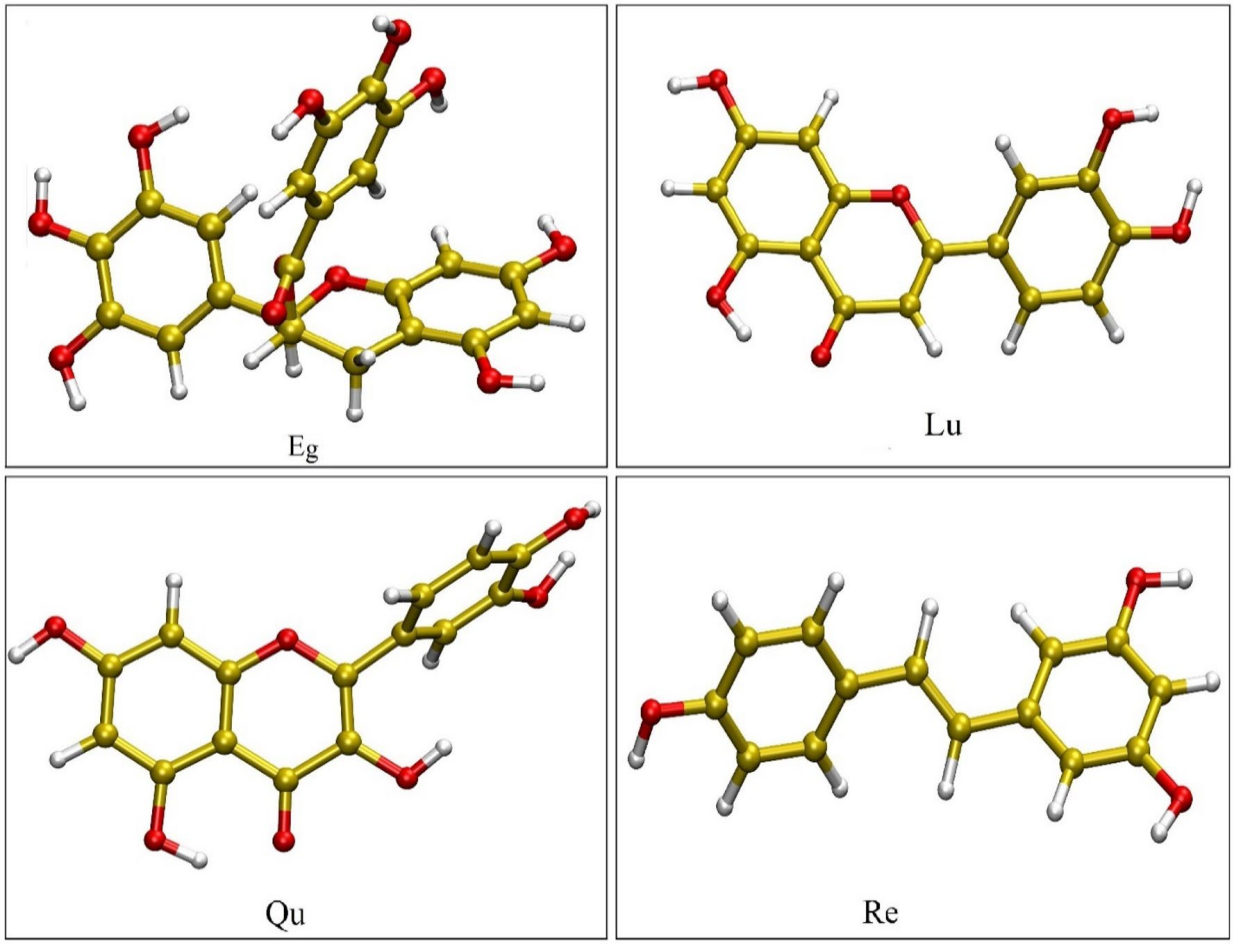
The chemical structures of Epigallocatechin-3-gallate (EG), luteolin (Lu), quercetin (Qu), and resveratrol (Re).

The initial structure of PC (see Fig. 1b) is extracted from human phosphatidylcholine transfer protein which is taken from RCSB Protein Data Bank (PDB) (https://www.rcsb.org/, PDB ID: 1LN3)^36^. The chemical structures of polyphenol compounds are downloaded from the PubChem server (PubChem CID: EG = 65,064, Re = 445,154, Lu = 5,280,445, and Qu = 5,280,343)^37^. The chemical structures of the studied polyphenols are given in Figs. 2 and S1. It should be noted that all of the monomer molecules are fully optimized at the M062X/6-311G** level of theory^38^ using Gaussian 09 software^39^.

Figure S2 represents the map of the electrostatic potential (MEP) overlaid on 0.001 au isodensity surface of PC and PP molecules. The electrostatic potential intensifications are displayed on the color scale as: red (more negative) < orange < yellow < green < blue (positive). A prevalence of green shows the potential midway between the two extreme ends. This Figure confirms that the polar head of PC is more active than tail part.

### Molecular mechanics calculations

For the interaction of phospholipids with neutral products during the formation of phytosome, previous studies suggested that the HBs play a major role^26^. Due to the presence of several heteroatoms in the structures of the polyphenols and head group of PC, the HBs can form in different manners (see Fig. S2). The conformer search analysis is performed to find the most preferable conformers of Eg-PC, Lu-PC, Qu-PC, and Re-PC complexes. To design starting configurations, each of the studied polyphenols is placed near the PC polar heads so that they can form the most hydrogen bonds. Then, for each complex 1000 conformers are analyzed under the Merck mechanics force field (MMFF)^40^ using the Spartan package^41^. In this force field, according to molecular mechanics protocols, the out-of-plane bending and dihedral torsion parameters are changed to provide a better molecular geometry for molecules or complexes. From the outcome of this calculation, the lowest energy structures for Eg-PC, Lu-PC, Qu-PC, and Re-PC complexes are extracted and used as the starting configurations in the QM calculations.

### Quantum mechanical calculations

The QM calculations are performed on the extracted PP-PC complexes from MM calculations using the Gaussian 09 programs^39^. The global hybrid meta GGA (M06-2X) functional is used to optimize the complexes. This functional can simulates well the hydrogen bonds and nicely typifies other weak interactions. Several recent studies have demonstrated the reliability of the outcomes of this functional in many chemical and biological phenomena^38^. The 6‐311G** Gaussian basis set selected for optimization includes polarized function added on the heavier and hydrogen atoms. Normal vibrational frequencies are calculated by performing the single-point calculation on the optimized structures. All minima on the potential energy surface of the molecule were confirmed with the positive value of Hessian eigenvalues. The Gaussian output WFX file was subsequently subjected as an input to the AIMAll package^42^ to perform the quantum theory of atoms in molecules (QTAIM) analysis to shed light on the nature and strength of the intermolecular interactions in the studied complexes. Furthermore, the intermolecular interactions were further analyzed via the noncovalent interaction-reduced density gradient (NCI-RDG) method^43^ using the Multiwfn program^44^. In the QTAIM method RDG plot is determined through the following equation:

1 $$s=\frac{1}{2{\left(3{\pi }^{2}\right)}^\frac{1}{3}}\frac{\left|\nabla \rho \right|}{{\rho }^\frac{4}{3}}$$

where $\rho$ and $\nabla \rho$ are density and its first derivative.

Energy decomposition analysis (EDA) for the optimized DFT geometries is conducted using the ADF program^45^. To evaluate the potential orbital interactions between the polyphenols and PC, neutral bond orbital (NBO) analysis is performed using the NBO program^46^ as implemented in the Gaussian 09 package. It should be noted that for all of the post-QM analyses, single-point calculations were performed at the same level of theory as this used in the geometry optimizations.

### Molecular dynamics simulation

**Figure 3:**
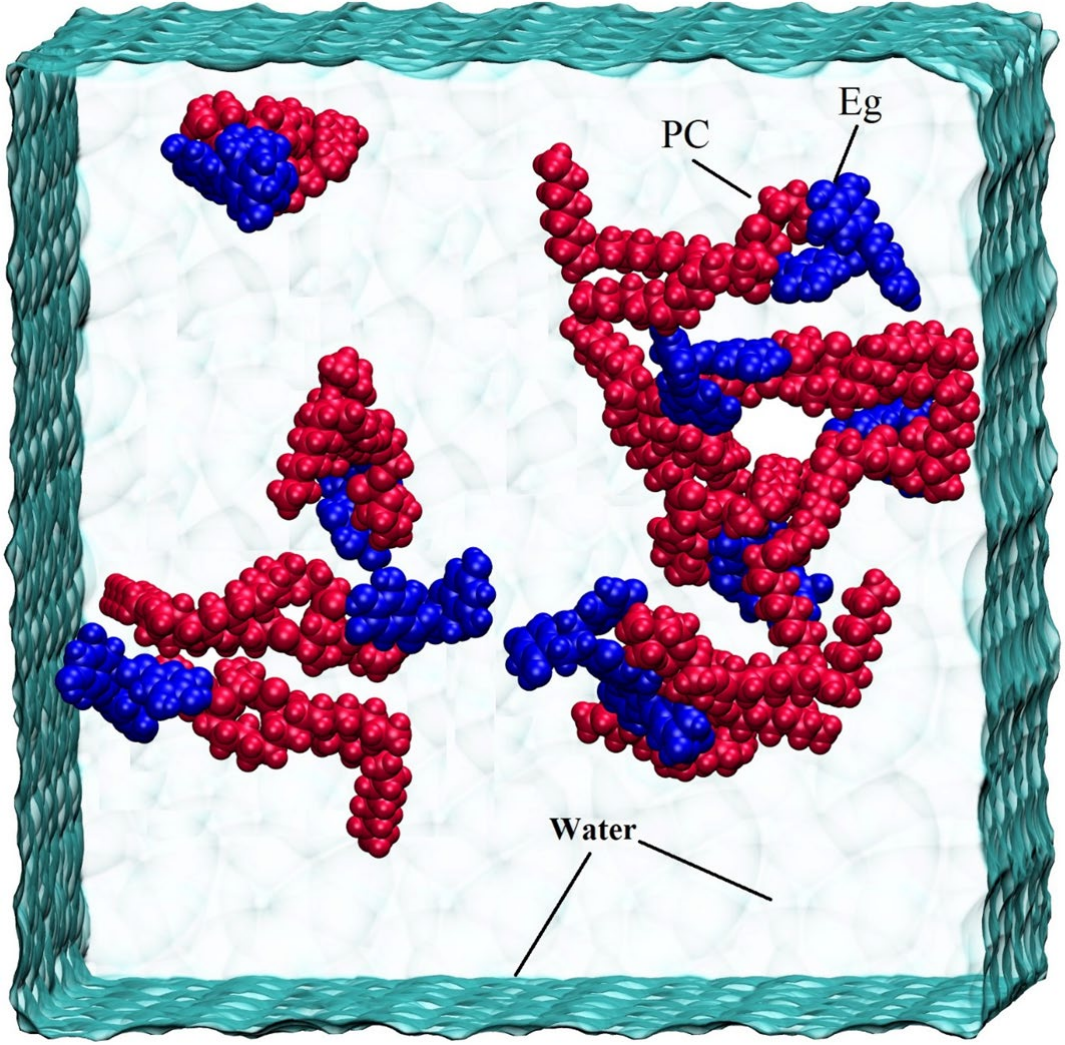
The initial snapshot of for Eg-PC system as a representative model of the designed MD simulation box.

To build the MD simulation models, the optimized geometries of PC-PP complexes are selected. Four simulation boxes (hereafter named Eg-PC, Lu-PC, Qu-PC, and Re-PC systems) are designed in which 10 molecules of PC-PP are randomly inserted into the boxes. Then, the simulation boxes are solvated using the three-site transferrable intermolecular potential (TIP3P) water model^47^. The Na^+^ and Cl^−^ ions are added for neutralizing the systems. This environment is select to mimic the bMore details about the simulation systems are provided in Table S1. For all components of systems, the force field parameters are applied by the CHARMM force field^48^. As an example, a snapshot from one of the simulation boxes is given in Fig. 3. In this study, the GROMACS package^49^, version 2021.3, is used to perform the MD simulation runs and post-MD analyses. The steepest descent algorithm^50^ is employed to apply 1000 steps of energy minimization.

For 500 ps, all of the simulation systems are subjected to two runs in NVT (constant number, volume, and temperature) and NpT (constant number, pressure, and temperature) ensembles. The temperature using V-rescale (V stands for velocity) thermostat^51^ slowly increased to 310 K. Also, by the Berendsen algorithm^52^ pressure reached 1 bar and is kept in this state during simulation. MD productions are run for 150 ns under periodic boundary conditions. All of the bonds restrain at heir equilibrium position with the LINear Constraint Solver (LINCS) algorithm^53^. The Longrange electrostatic interactions are treated by the particle-Mesh Ewald (PME) method^54^, and nonbonded interactions are calculated with a 1.2 nm range cutoff.

### Steered molecular dynamics simulations

**Figure 4:**
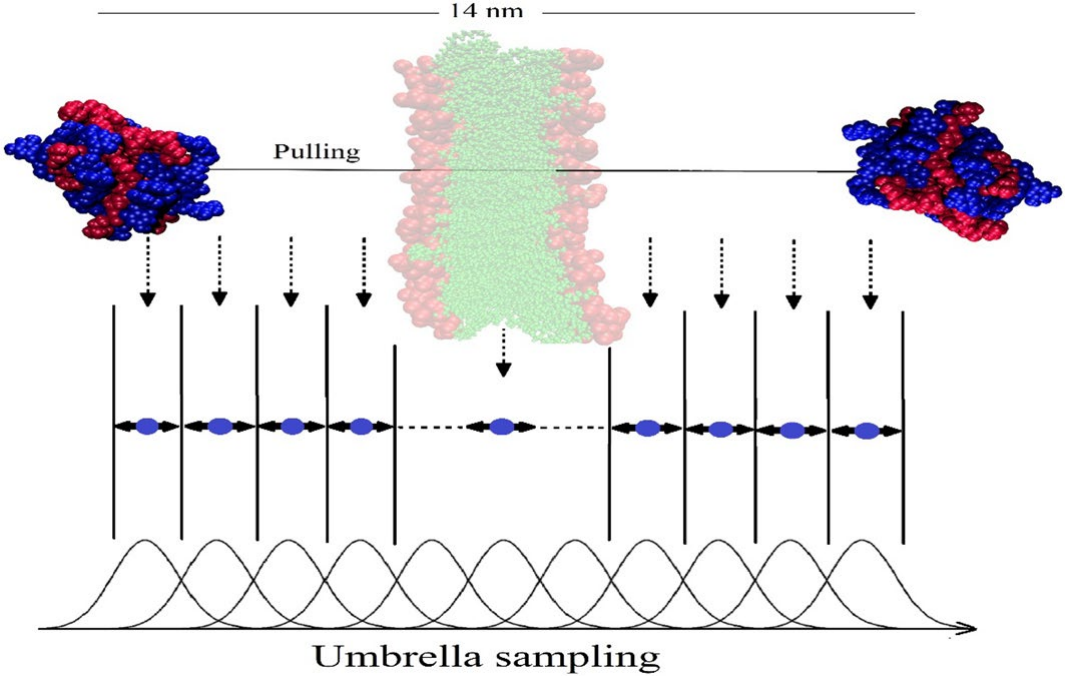
Illustration of the (top) pulling simulation of phytosome through the cell membrane and the (bottom) the umbrella sampling simulation/weighted histogram analysis method.

As mentioned in the “Introduction” section, the great improving the membrane permeability of NPs is one of the main benefits of phytosome. Thus, the SMD simulations are performed to assess the penetration of the studied phytosomes across the cell membrane. All the details of our membrane model are similar to our recent work^55^. In SMD simulations, four individual boxes with the dimension of 28 × 11 × 11 nm^3^ are designed. The phytosome complexes are located on the top of lipid bilayer at a distance of around 6 nm. These boxes are filled with TIP3P water and neutralized with Na and Cl ions. The phytosome is pulled about 14 nm in the simulation box that passed from the membrane (Fig. 4). This is performed using the pull code option available on the GROMACS software. The pulling process and Umbrella Smpiling are done according to the following protocol. The energy minimizing using steepest descent method is performed to reduce the net forces on atoms in the simulation boxes. This is followed by equilibrated steps to maintain the systems at temperature and pressure of 310 and 1 bar, respectively. The Nose–Hoover thermostat^56^ is employed to keep the temperature, and the Parrinello-Rahman barostat^57^ utilized to semi-isotropically control the pressure. The phytosome subjected to the external force (2000 kJ/mol nm^2^) with rate of 0.5 nm/ns to cross the membrane. The force between the center of mass (COM) of the phytosome and the COM of the membrane is computed by the following equations:

2 $$U=\frac{1}{2}k{\left[vt-\left(r-{r}_{0}\right) \cdot n\right]}^{2}$$

3 $$F=-\nabla U$$

where U is the potential energy, k, v, t, r, r_0_, and n is the spring force constant, pulling speed, the current time, the position vector of the COM of phytosome at t, the initial position vector of the COM of phytosome, and the unit vector indicating the direction in which the dummy atom is pulled, respectively. The z-axis (the direction of diffusion into the membrane) is chosen as a pulling direction. The SMD simulations for 2.8 ns are performed to generate the initial configurations for equal spaces (0.25 nm) “windows” of the Umbrella Sampling simulations (see Fig. 3). The weighted histogram analysis method (WHAM) is used to calculate the potential of mean force (PMF) from the Umbrella Sampling simulations.

## Results and discussion

### DFT calculations

**Figure 5:**
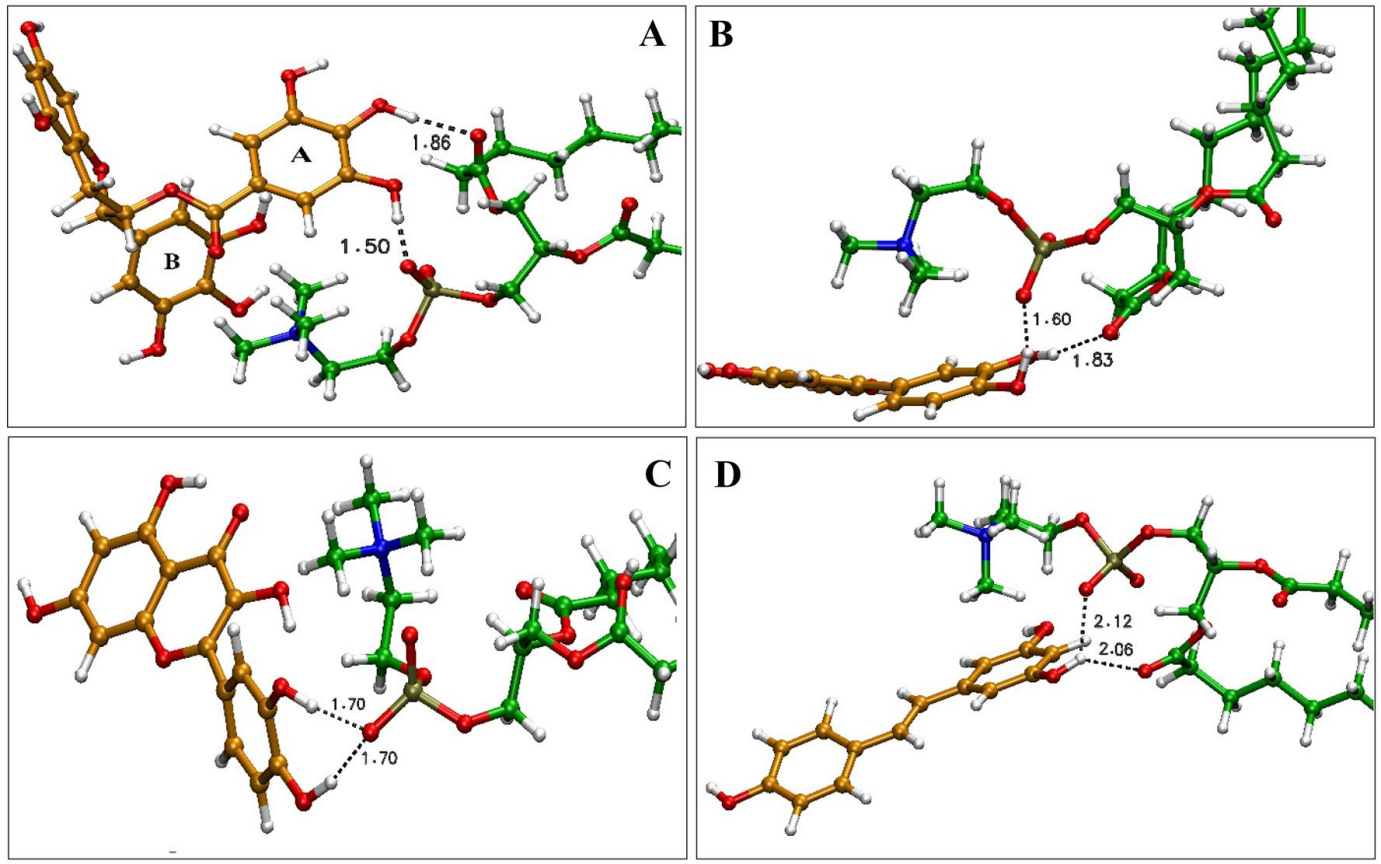
Close-up snapshots from interaction sites of the optimized structure of (**A**) Eg-PC, (**B**) Lu-PC, (**C**) Qu-PC, and (**D**) Re-PC complexes. Color code; C in PP: orange, C in PC: green, O: red, N: blue, P: brown, and H: white.

Energy minimal optimized geometrical configurations of the studied complexes are given in Fig. 5. As can be seen in Fig. 5A, several intermolecular interactions are formed between the Eg and PC. These interactions can be divided into two following types;

i. Two strong intermolecular HBs of the phosphate and glycerol parts of PC with hydroxyl groups of A ring in the Eg molecule
ii. Several partial HBs (including CH…O and CH…pi) interactions of the choline part with the B ring of the drug (see Fig. 5).

It seems that HBs are stronger than the other interactions where the intermolecular distances in type *“i”* are closer than that in type *“ii”.* This observation will be further explored in the QTAIM section. Interestingly, it is observed that due to the high affinity of Eg to form the complex, some of the drug angles and dihedrals are changed during complex formation. As shown in Fig. 5B, the Lu behaves similarly to EG in interaction with the phospholipids in which the OH…O, CH…O, and CH…pi govern the major role in the stability of the Lu-PC complex. It is worth mentioning that the HB intermolecular distances in this complex are farther than that in EG-PC. This observation could be attributed to the planer structure of Lu, which restricts the changes in its orientation. In other words, due to the trident structure of the Eg molecule, it can be easily reorientated and form stronger HBs. Qu for interaction with PC shows different behavior from the Eg and Lu drugs. As can be seen in Fig. 5C, two hydroxyl groups of Qu molecule form HBs with the phosphate part of PC. Furthermore, the intermolecular distances in these interactions are farther than the Eg and Lu. It should be noted that the CH…pi interaction in this system is disappeared, which indicates a lower tendency of Qu drug to interact with phospholipids. In the Re-PC system (Fig. 5D), the HB distances between the donor and acceptor groups are much farther than in the rest systems. However, the role of CH…pi interaction in this complex is more prominent. Overall, it can be concluded that the intermolecular HBs of phosphate and glycerol parts of PC with the polyphenol compounds are the main driving force in the formation of phytosomes. Also, the vdW interaction such as CH…O and CH…pi interaction between the choline part and drug reinforce the HB interactions.

**Figure 6:**
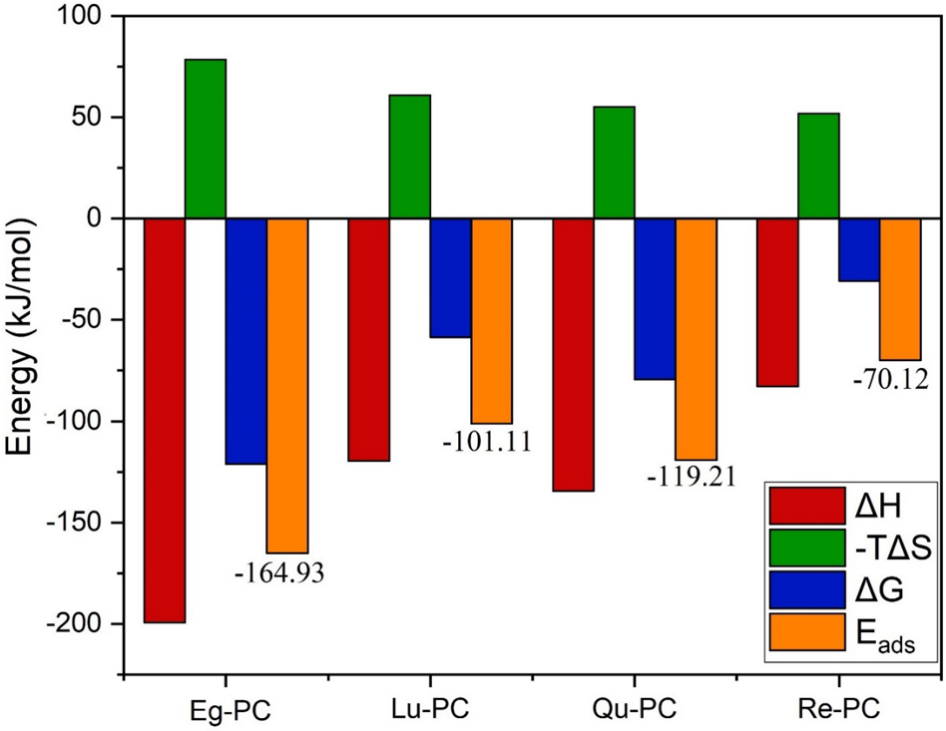
Thermodynamic parameters and adsorption energy of the studied complexes.

The adsorption energy (E_ads_) of the studied complexes is obtained from the supramolecular approach, where the adsorption energy is equal to the difference between the total electronic energy of the monomers and the complex, taking into account the basis set superposition error (BSSE). The obtained energy results are reported in Fig. 6. The Eads values in the investigated systems are negative and varied from − 70.121 to − 164.928 kJ/mol. The negative values confirm that the formation of phytosomes between the studied PPs and PC is energetically favorable.

The Gibbs free energy (∆G) parameters are calculated to scrutinize the thermodynamic parameters during the formation of the phytosomes (Fig. 6). The ∆G values for all of the PP-PC systems are < 0, which shows this process is spontaneous and exergonic. It should be noted that the lowest Gibbs free energy belongs to the Eg-PC complex, which confirms this complex is the most stable phytosome. This result can be attributed to the formation of strong intermolecular interactions between Eg and PC molecules. The hierarchy of Gibbs free energy terms follows the order of Eg-PC > Qu-PC > Lu-PC > Re-PC. The comparison of the Gibbs free energy trend with the reported intermolecular distance (Fig. 5) shows that the system with the closer intermolecular distance has higher Gibbs free energy. Close inspection of thermodynamic parameters indicated that, as expected, the entropy term in all of the systems is negative, and the most entropy belongs to the most stable complex. This is not a surprising result and reflects the fact degree of freedom of the system upon the formation of complexes is decreased. According to the entropy formula S = kLnW, where k and W stand for Boltzmann constant and the number of real microstates of the system, respectively. On the other hand, in statistical mechanics, W is dependent on the degree of freedom. Therefore, upon the complexion, by the decrease in the degree of freedom, the entropy of the systems is reduced.

The EDA is a powerful method that provides a conceptual interpretation of the nature of the interaction between two fragments. Based on Morokuma’s bond energy decomposition method^58^, the EDA decomposes interaction energy (ΔE_int_) between two fragments in the complex into three well-defined terms:

4 $$\Delta Eint= \Delta {V}_{elst}+ \Delta {E}_{pauli}+\Delta {E}_{orbit}$$

**Figure 7:**
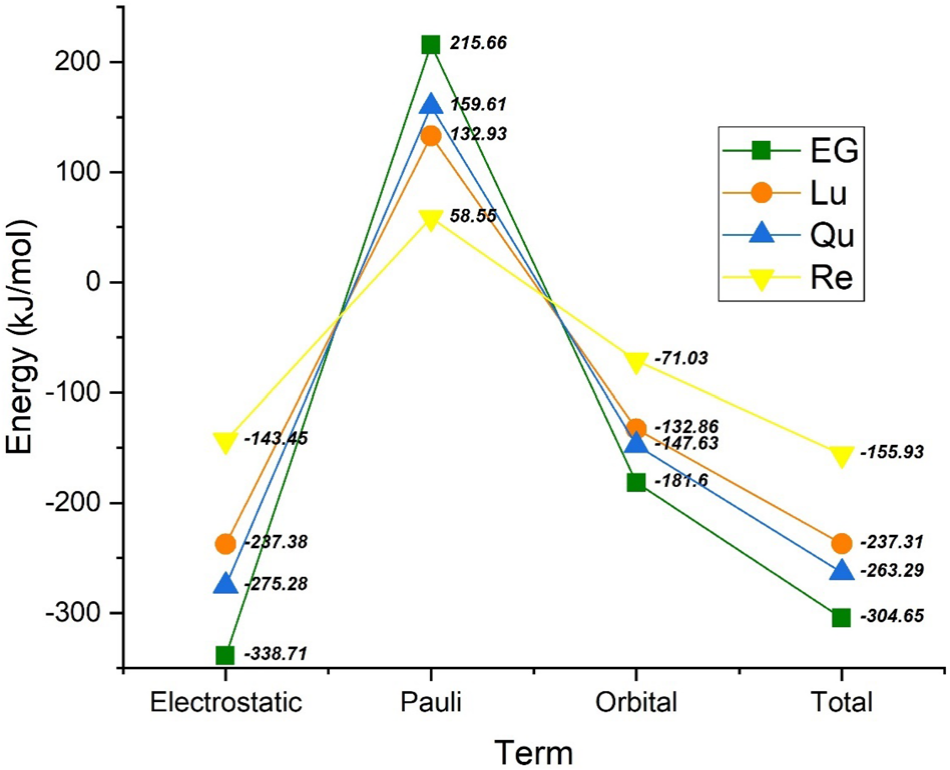
Energy decomposition of the interaction energy of the complexes into Pauli repulsion (E_Pauli_), electrostatic (E_elst_), and orbital interaction (${E}_{orbit}$).

These physically meaningful terms are (i) quasiclassical electrostatic interaction energy (ΔV_elst_) between the unperturbed charge densities of the prepared fragments (ρ_PC_ + ρ_PP_), (ii) Pauli repulsion (ΔE_pauli_) between the fragments that arise from Pauli’s principle, and (iii) orbital interaction energy (ΔE_orbit_) that accounts for electron pair bonding, charge transfer, and polarization. Figure 7 shows the obtained results of EDA calculation for the studied systems. As can be seen in this Figure, the electrostatic interactions (hydrogen bond and dipole-diploe interactions) have a major contribution to the stabilization of the studied complexes. The Pauli repulsion is a positive term in which it in all of the complexes is larger than the orbital interaction energy. It means that the strong interaction energy arises from the quasiclassical electrostatic interaction. The order of the energy terms is in good agreement with the adsorption energy results and shows that the EG-PC system is the most stable complex. Furthermore, the comparison of two attractive terms demonstrates the contribution of E_elst_ in interaction is more than the orbital term. This finding indicates that the formation of the phytosome has non-covalent nature, which is in line with the experimental findings^59^.

The intermolecular interactions are further analyzed using Bader’s QTAIM and the RDG methods. These analyses detect interactions that cannot be measured by experimental methods such as X-ray data analysis^60^. The obtained results from QTAIM and RDG calculations are summarized in Table 1 and Figs. S3–S6. Numerical data of the topological parameters including electron density (ρ), Laplacian of electron density (∇^2^ρ), potential (V), kinetic (G), and total (T = V + G) electron energy densities are obtained in the intermolecular bond critical points (BCPs).

**Table 1:** The electron densities (ρ), its Laplacian (∇^2^ρ), kinetic electron density (G), the local potential electron energy density (V) and the total electron energy density (H) (all in a.u.), the hydrogen bond energy (E_HB_, kJ mol^−1^) between PPsand PC.

System	Pair	ρ	∇^2^ρ_BCP_	V_BCP_	G_BCP_	H_BCP_	E_HB_
Eg	**O22–H184**	**0.07194**	**0.17029**	**− 0.08282**	**0.06269**	**− 0.02012**	**− 108.718**
	O41–H185	0.02681	0.10405	− 0.02206	0.02404	0.00198	− 28.9632
	H14–O141	0.00961	0.03386	− 0.00603	0.00725	0.00122	–
	H10–O141	0.0135	0.04833	− 0.00869	0.01039	0.00169	–
	H12–O141	0.01091	0.03454	− 0.00681	0.00772	0.00091	–
	H38–O144	0.00575	0.0237	− 0.00413	0.00503	0.00090	–
	H13–O139	0.00664	0.0255	− 0.00449	0.00543	0.00094	–
	H13–O142	0.01176	0.03893	− 0.00752	0.00862	0.00111	–
	H16–O140	0.00873	0.03072	− 0.0057	0.00669	0.00099	–
	H12–C163	0.00768	0.02481	− 0.00405	0.00512	0.00108	–
	H16–C160	0.00804	0.02963	− 0.00466	0.00603	0.00138	–
	H12–C159	0.00725	0.02455	− 0.00386	0.005	0.00114	–
Lu	**O22–H165**	**0.05315**	**0.17181**	**− 0.05837**	**0.05066**	**− 0.00771**	**− 76.6265**
	O41–H164	0.03076	0.11761	− 0.02671	0.02806	0.00135	− 35.0609
	C42–O139	0.00674	0.02787	− 0.00455	0.00576	0.00121	–
	H10–C154	0.00553	0.01392	− 0.0025	0.00299	0.00049	–
	H12–C144	0.01107	0.03584	− 0.006	0.00748	0.00148	–
Qu	**O21–H165**	**0.04466**	**0.15002**	**− 0.04544**	**0.04147**	**− 0.00397**	**− 59.6566**
	**O21–H166**	**0.04084**	**0.15436**	**− 0.04195**	**0.04027**	**− 0.00168**	**− 55.0646**
	H7–O136	0.01177	0.04519	− 0.00796	0.00963	0.00167	–
	H16–O136	0.00833	0.02863	− 0.0052	0.00618	0.00097	–
	H13–O136	0.01181	0.0408	− 0.00752	0.00886	0.00134	–
	H11–O140	0.0049	0.01663	− 0.0029	0.00353	0.00062	–
	H18–C154	0.01037	0.03312	− 0.00579	0.00703	0.00125	–
Re	O22–H162	0.01814	0.06902	− 0.01426	0.01576	0.0015	− 18.7172
	O41–H162	0.0171	0.07366	− 0.01328	0.01585	0.00257	− 17.4320
	O41–H158	0.00688	0.02243	− 0.00422	0.00491	0.00069	–
	H18–C148	0.00936	0.02658	− 0.00468	0.00566	0.00098	–
	H11–C145	0.01085	0.0389	− 0.00623	0.00798	0.00175	–

The strong hydrogen bonds show in bold font.

The QTAIM results showed that the value of ρ at BCP and the number of the formed interatomic interactions in the Eg-PC complex are more than in the rest complexes. Furthermore, in all of the systems, the value of electron densities for the OH…O hydrogen bond interaction is much higher than the other intermolecular interactions strength of interactions is evaluated using the Rozas et al. method^61^, in which the interactions are classified based on values and signs of H_BCP_ and ∇^2^_ρBCP_. The electron density values for HBs are within the range of 0.0171–0.0719 a.u., which is lower than covalent bonds. H_BCP_ and ∇^2^_ρBCP_ for all of the interatomic interactions are positive except for the strong hydrogen bonds (in bold font in Table 1), which indicates the weak and medium strength of these interactions. While for the strong HBs, the ∇^2^_ρBCP_ is still positive, the negative V_BCP_ value more than the positive value of G_BCP_ results in a negative H_BCP_, suggesting this hydrogen bonding is rendered with partial covalent nature.

In each of the systems, two HBs are formed, and the total energy of these hydrogen bonds obtained from the Spinoza method in each of the systems follows the order of Eg-PC > Qu-PC > Lu-PC > Re-PC. This order has good agreement with the adsorption energy trend and precisely shows that hydrogen bonding is a major factor in the stability of PP-PC complexes. The number of BCPs in the Eg-PC system is more than in the other complexes where 12 BCPs between Eg and PC are formed. It can be attributed to the flexible structure of Eg, which is relatively easily reoriented and form a more stable complex. Thus, it can be stated that the flexibility of the PP molecules is an important factor in the formation of intermolecular interactions (HB or vdW interactions) in the phytosome. It should be noted that the lowest ρ values and consequently the weakest HBs belongs to the Re-PC system.

**Figure 8:**
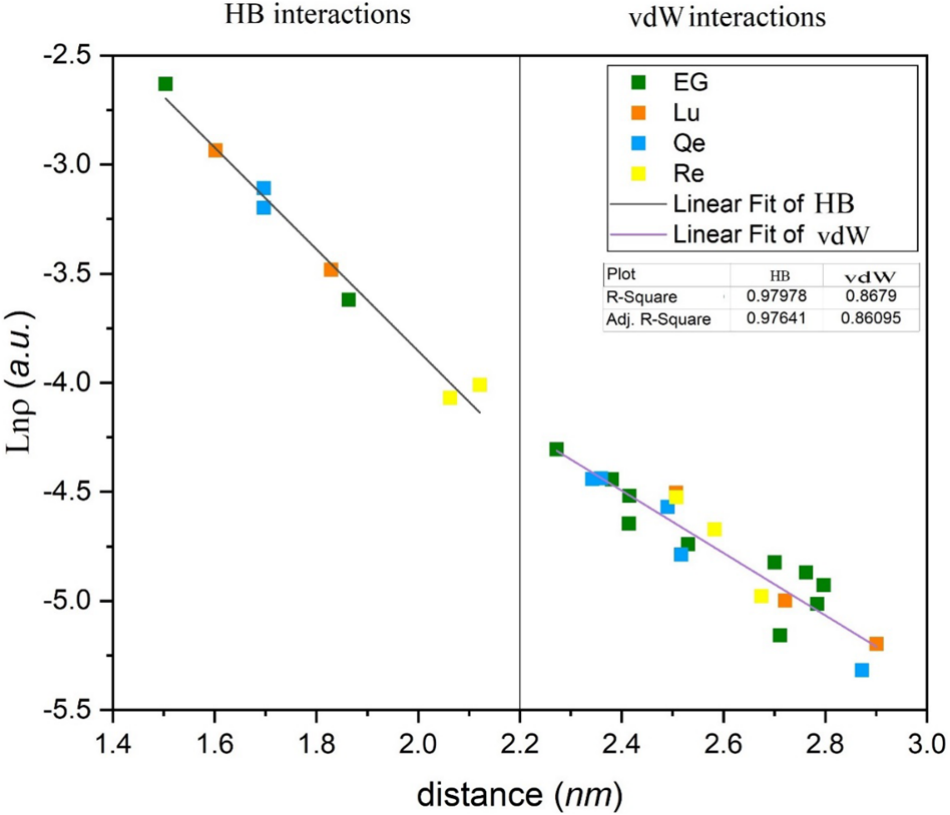
ln(ρ_BCP_) (left: hydrogen bonds and rigth: van der Waals regions) as a function of intermolecular distance in the investigated complexes.

The plot of ln(ρ_BCP_) vs. the interatomic distances for the HB and vdW interactions is shown in Fig. 8. This plot deciphered the correlation of the electron density with the interatomic distances. The correlation coefficients for linear fit at the HB and vdW regions are ~ 0.97 and 0.89, respectively. Accordingly, it can be concluded that there is a reciprocal relation between electron density and interatomic distances. Furthermore, the lower correlation coefficients for vdW interactions indicate more change arising from the formation and deformation of these interactions during adsorption. While, hydrogen bonds are relatively strong, which leads to a higher correlation coefficient.

**Figure 9:**
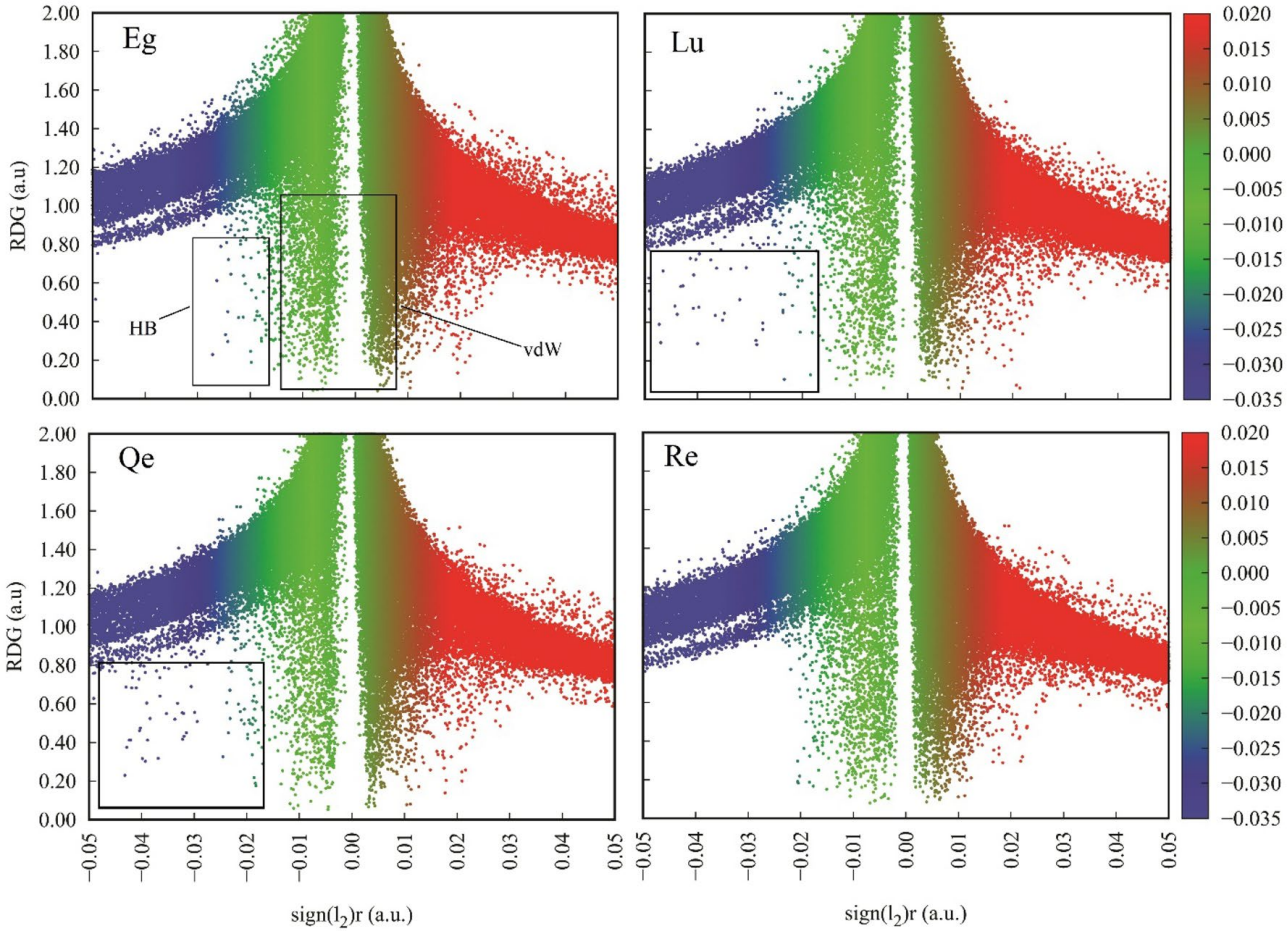
Color-filled RDG isosurfaces and the RDG vs sign (λ_2_)ρ plots the studied complexes.

The NCI‐RDG is a technique based on BCP described through the QTAIM theory and is used to provide deeper insights into the nature of PP-PC interactions. The importance of the NCI method is its ability to elucidate attractive, repulsive, steric, and weak dispersive interactions in real space and as a graphical representation. The outcomes of this method provide the fingerprint region of close contact within the intermolecular interactions. The 2D plots of the reduced density gradient versus the electron density multiplied by the sign of the second Hessian eigenvalue (λ2) for the PP-PC complexes are given in Fig. 9. The 3D spatial visualizations of NCI isosurfaces for the studied complexes are depicted in Figs. S3–S6. The emergence of a small and flat blue isosurface between one of the O–H···O site in the Eg-PC complex (HB2 in Fig. S3) confirms the earlier inferences on the hydrogen bonding. Interestingly, for the strongest hydrogen bond (HB1 in Fig. S3), the blue region does not appear. Consequently, although two strong hydrogen bonds are formed in this system, the 2D plot does not show the formation of strong hydrogen bonds (Fig. 9A). The van der Waals (vdW) interactions that formed between Eg and PC appear as the greenish sheet‐like region in the NCI isosurface (see Fig. S3). In the 2D RDG plot of the Eg-PC system, the green area is considerable, which can be attributed to the formation of several vdW interactions in this complex. In the Lu-PC and Qu-PC complexes, the presence of two OH…O hydrogen bonds leads to the appearance of two small, flat, pill-shaped blue regions in their isosurfaces (Figs. S4, S5). It should be noted that these hydrogen bonds can be detected by the 2D RDG plot where the blue points emerge. Furthermore, as can be seen in Fig. 9, the vdW interactions in these systems are much lower than in the EG-PC and Re-PC systems. In the Re-PC complex, the vdW interactions play the main role in the stability of the phytosome, therefore, the green area in its isosurface and the 2D plot emerges.

Overall. according to the DFT calculation, it can be concluded that in addition to hydrogen bonding, van der Waals interactions also play a significant role in the stability of phytosome.

### MD simulations

**Figure 10:**
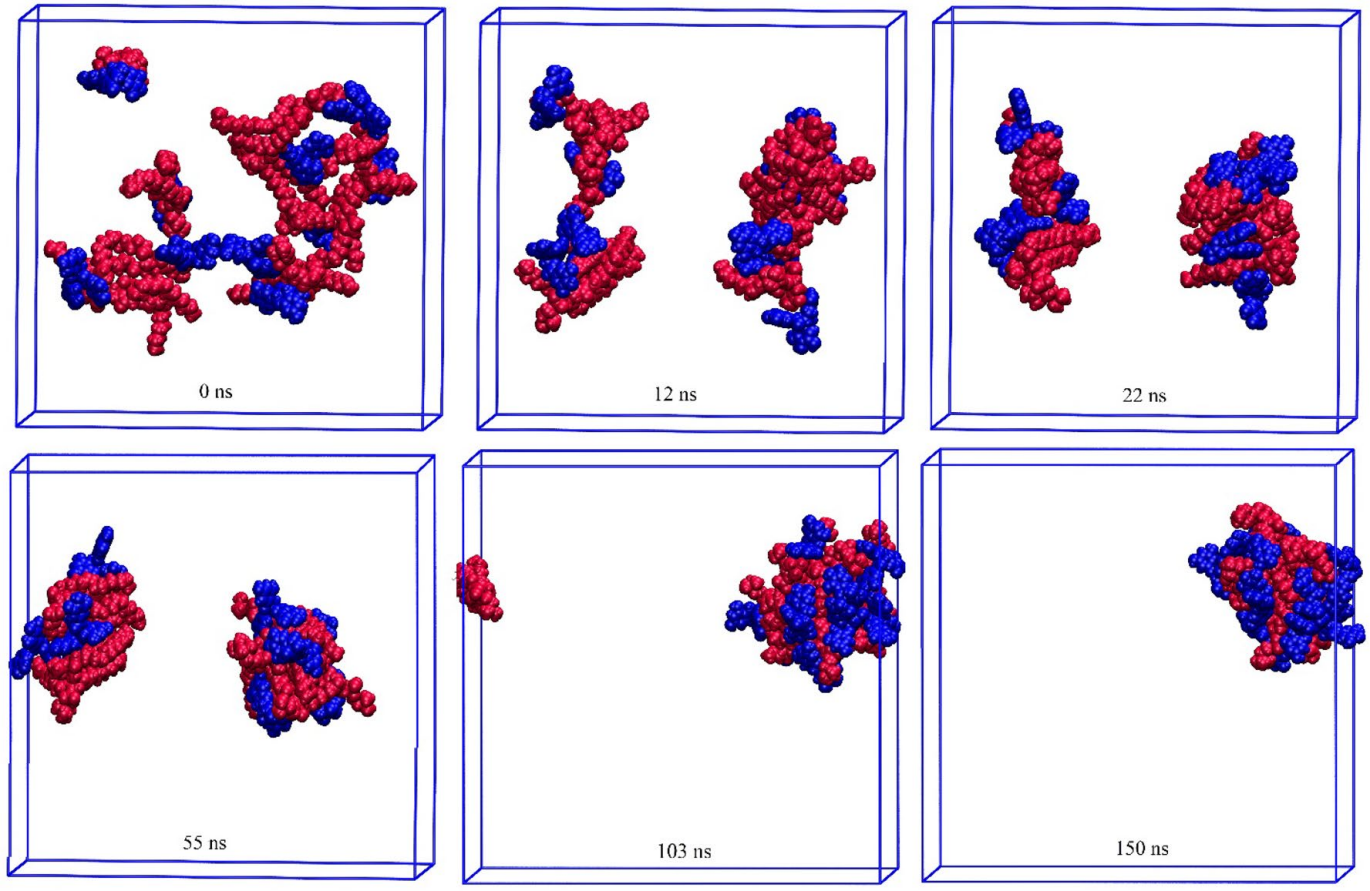
Self-aggregation process of Eg-PC monomers into a phytosome as a function of MD simulation time. Color code; EG: blue and PC: red.

**Figure 11:**
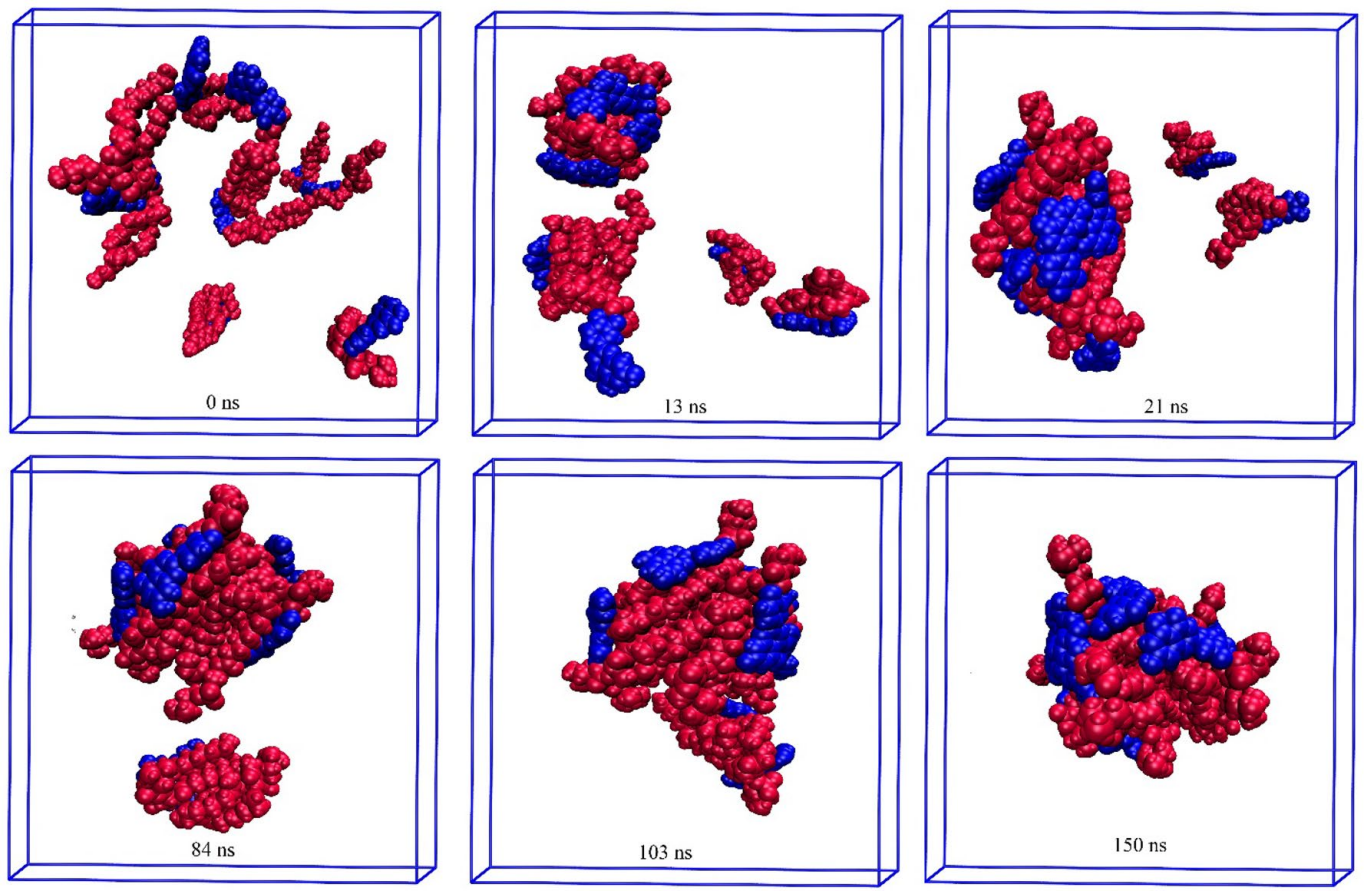
Self-aggregation process of Lu-PC monomers into a phytosome as a function of MD simulation time. Color code; Lu: blue and PC: red.

**Figure 12:**
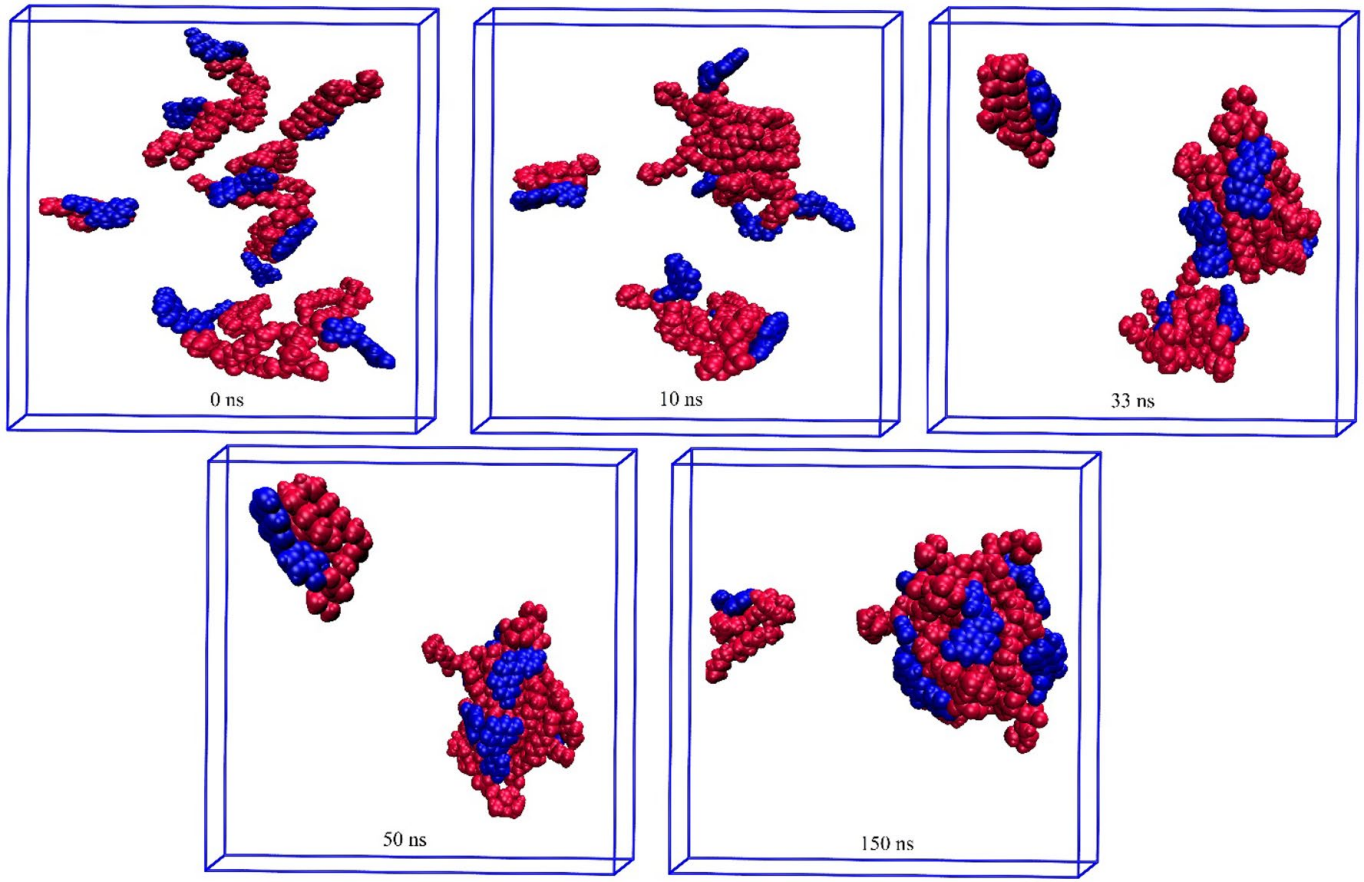
Self-aggregation process of Qu-PC monomers into a phytosome as a function of MD simulation time. Color code; Qu: blue and PC: red.

**Figure 13:**
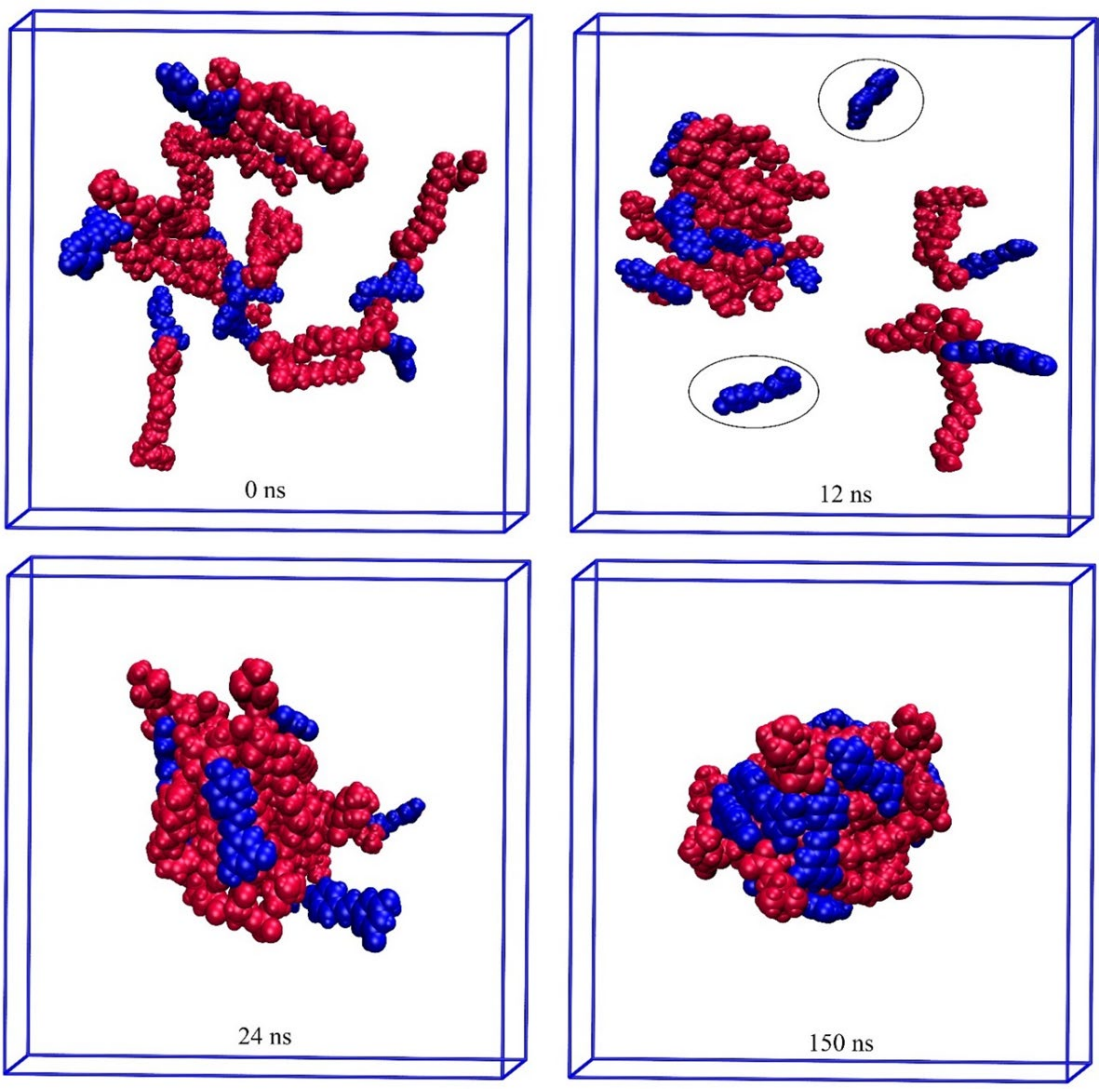
Self-aggregation process of Re-PC monomers into a phytosome as a function of MD simulation time. Color code; Re: blue and PC: red.

In this section, a series of all-atoms MD simulations are performed to evaluate and analyze the phytosome formation process. The details of the designed simulation boxes are provided in the “The Strategy of Calculations” section. In Figs. 10, 11, 12, 13, the timescale for the self-aggregation of phytosome from the random position of their initial configurations is depicted. In the Eg-PC system, after 12 ns, two small aggregate molecular clusters are formed. As a function of simulation time, the formed clusters become more compacted so that at around 55 ns, their shape turn to relatively spherically. Then, as shown in Fig. 10 the two clusters approach each other, and eventually, at 108 ns, the phytosome begins to form. The final snapshot of the Eg-PC system confirms that a stable phytosome is formed.

The Lu-PC system behaves almost similarly to the Eg-PC system, where two clusters are formed approximately after 13 ns (Fig. 11). However, as can be seen in Fig. 11, the two Lu-PC molecules remain separate. At 21 ns, two small clusters are joined together and are turned into a bigger one. It should be noted that the two separated molecules become closer and interact with each other. The Lu-PC molecules at 84 ns become closer, and finally, the phytosome is formed at 103 ns. At the end of the simulation, the phytosome of Lu-PC is compacted, which confirms the Lu-PC, like Eg-PC, potentially tends to form a stable phytosome.

In the Qu-PC system, initially (10 ns), two small clusters are formed, which at 33 ns are joined together (Fig. 12). Interestingly, it is observed that one of the Qu-PC molecules remains separately from 10 to 150 ns of MD production. Accordingly, it can be concluded that despite the DFT calculations showing that there is a strong interaction between Qu and PC (adoption energy = − 119.21 kJ/mol), this system has a lower tendency to form phytosome than the two former systems. In other words, in a phytosomal system, the interaction between its components can be strong enough, but the tendency to form a stable phytosome is less.

The impact of the intermolecular interaction between the two constituents of the phytosome is evident in the Re-PC system (Fig. 13), where the Re-PC complex is unstable and degraded in the presence of explicit TiP3P water molecules. However, at the end of the simulation, the drug and phospholipid molecules are self-assembled together and form a spherical complex. It should be noted that the intermolecular interactions between the drug and PC in Eg-PC, Lu-PC, and Qu-PC systems remain stable in the whole of MD production. By comparing the obtained results from the DFT calculations and MD simulations, it can be concluded that hydrogen bonding is the main driving force in the stability of PP-PC complexes. In the other words, the formation of strong hydrogen bonds (H_BCP_ < 0 and ∇^2^_ρBCP_ > 0) protects the complex from degradation in the aqueous environment.

Furthermore, it is found that in all of the studied systems, the aliphatic chains of PC are compressed in the presence of water molecules. Also, as shown in Figs. 10, 11, 12, 13, the drug molecules and the hydrophilic part of PC are placed in the outer layer of the phytosome. This fact can be attributed to the presence of heteroatoms in their structures, which can form hydrogen bonds with water molecules.

After a qualitative study of the systems, in following a quantitative investigation of the systems will be done below.

The interaction energy between the PP-PC molecules for the studied systems is also calculated. The change in L-J interaction over the simulation time for PP-PC/PP-PC pairs is depicted in Fig. S7. The L-J energy values in all of the studied systems have decreasing trends indicating that the formation of phytosomes is a spontaneous process. The lowest L-J energy has belonged to the Eg-PC system, which confirms the high affinity of the Eg-PC molecule for the formation of phytosome.

**Figure 14:**
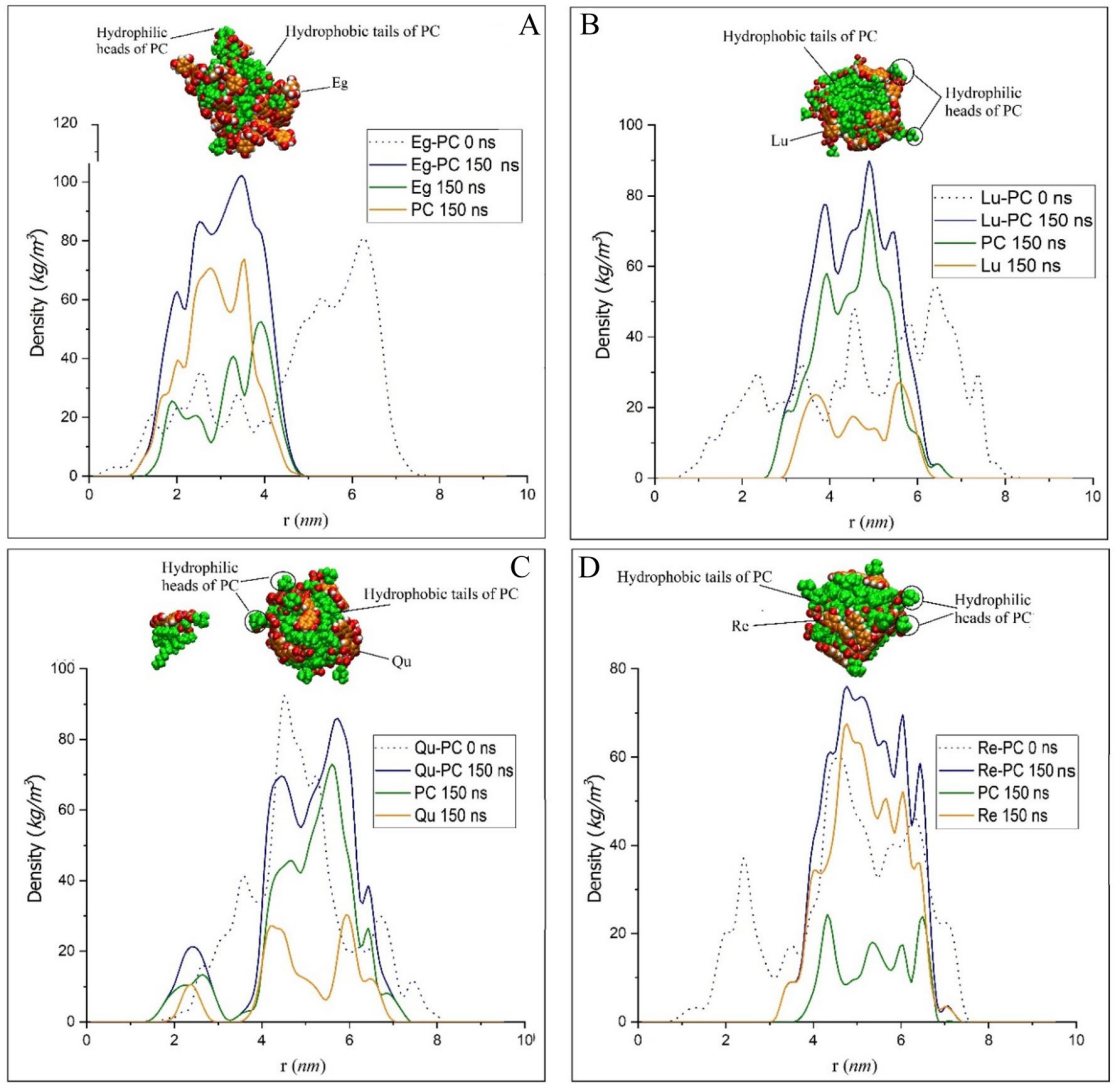
The density profiles for PP-PC complexes (at 0 ns and 150 ns), PP (at 150 ns), and PC (at 150 ns). (**A**) EG-PC, (**B**) Lu-PC, (**C**) Qu-PC, and (**D**) Re-PC systems.

The partial density profile of the studied systems is calculated by averaging the density values of the components of systems at intervals of 10 ps during the initial and final 2 ns of MD productions. The density profiles for the PP-PC (at 0 and 150 ns), PC (at 150 ns), and PP (at 150 ns) are given in Fig. 14. As observed in Figs. 10, 11, 12, 13, the PP-PC complexes at the start of MD productions are distributed inside the simulation boxes. This observation can be evidenced by the density profile plots in Fig. 14. As the molecules aggregate and the phytosome is formed, the amplitude of the density profile decreases, and its intensity increased. Since, the shape of the phytosome is almost spherical, its size can be determined based on the width of the peak in density profiles. Accordingly, Eg-PC, Lu-PC, Qu-PC, and Re-PC phytosome are 2.41, 2.78, 2.67, and 3.00 nm in diameter, respectively. It is found that the formed phytosome between Eg and PC is more compact than the rest PPs. As mentioned previously, the drugs and heteroatoms of PC orient in such a way positioned in the outer layer of the phytosome. This behavior can be confirmed by decomposing of density profile where the density profile of the drugs is more intense on two sides of phytosomes (see Fig. 14). According to obtained results, it is found that the hydrophobic tails of PC form the core of the phytosome and are wrapped by drugs and hydrophilic parts. The vdW interactions between the hydrophobic chains (It can be seen in RDG plots, Figs. S2–S5) create the core structure while exposing hydrophilic groups to the aqueous solvent. Therefore, it is evidenced that the lipid parts act as a nucleator. It should be noted that in the Qu-PC system (Fig. 14C), the density curve has an extra peak at 1.5–3.0 nm, which is related to the spared Qu-PC molecule.

The radius of gyration (Rg) can be used for measuring structure compactness, and it is useful to characterize polymer solutions and proteins. Based on the Rg, the formula (Eq. 5) is changed by varying the position of atoms (ri) with respect to the center of mass of the molecule.

5 $${R}_{g}={\left(\frac{{\sum }_{i}{\left|\left|{r}_{i}\right|\right|}^{2}{m}_{i}}{{\sum }_{i}{m}_{i}}\right)}^{0.5}$$

Here m is the mass of atom i. Furthermore, this parameter can be calculated around the coordinate axis. The following equation shows how the Rg can obtain for the x-axis.

6 $${R}_{g,x}={\left(\frac{{\sum }_{i}{{(r}_{i},y}^{2}+{r}_{i},{z}^{2}){m}_{i}}{{\sum }_{i}{m}_{i}}\right)}^{0.5}$$

**Figure 15:**
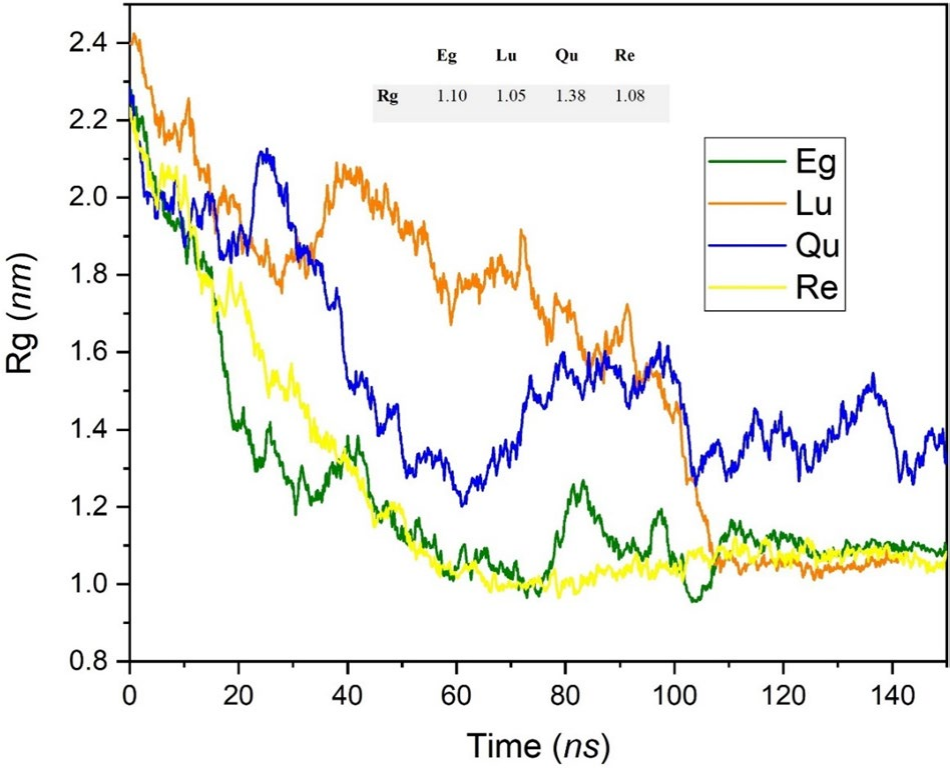
Radius of gyration (Rg,x) of the investigated complexes as function of simulation time.

A low Rg value shows the molecule structure is more compact and vice versa. The variation of Rg,x as a function of simulation time for the studied complexes is depicted in Fig. 15. The decreasing trend in Rg plots indicated that as a function of simulation time, the PP-PC complexes become more compact and confirm the formation of phytosomes. The fastest fall in Rg value has belonged to the Re-PC system, where the drug has the weakest interaction with PC, and their complex is destroyed in the presence of water molecules. Close inspection of Fig. 15 reveals that at the end of MD productions, the Rg plots for Eg-PC, Lu-PC, and Re-PC systems approximately reached the same values (about 1.1 nm), while for the Qu-PC system, the Rg is higher (around 1.4 nm) and has more fluctuation. This observation in the Qu-PC system could be attributed to the fact that one of the Qu-PC molecules remains individually in the aqueous medium.

**Figure 16:**
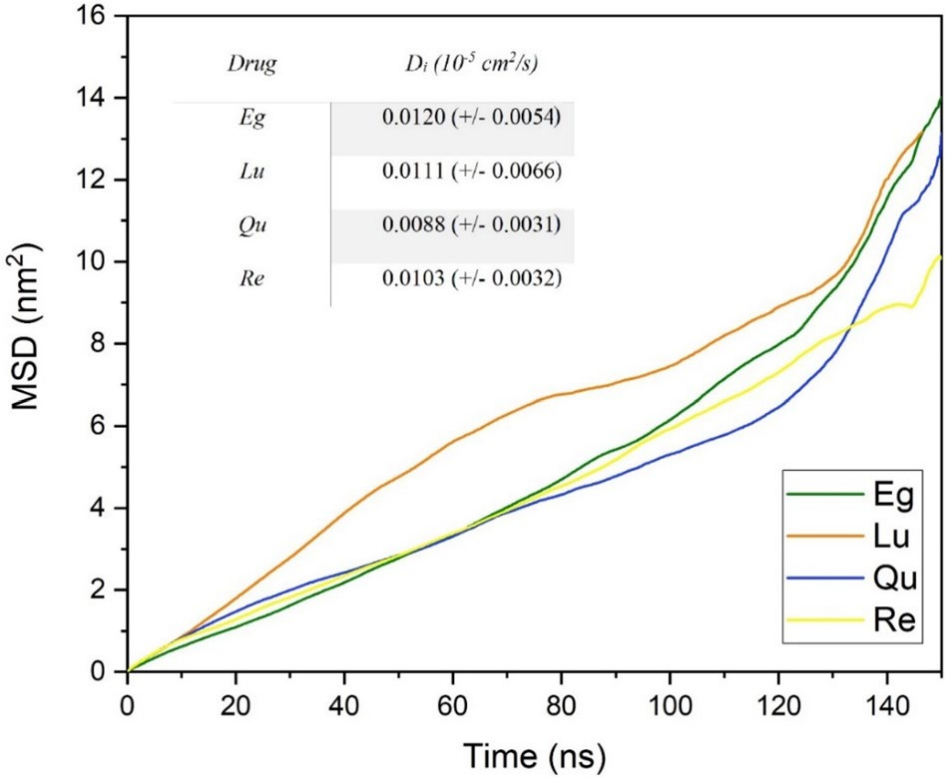
Mean square displacement of PP molecules in the investigated systems. Average diffusion coefficient (D_i_) of PPs shown in the table.

The mobility of drug molecules can be measured by evaluating the mean-square displacement (MSD). The self-diffusion coefficient (D) can also be computed from MSD based on the Einstein equation. In Fig. 16, the MSD plots as a function of MD production time for the investigated systems are illustrated. As can be seen in this Figure, the MSD curve of Eg-PC, Lu-PC, and Qu-PC systems have the same trend, and two regions with different slope are observed. Regarding the provided snapshots (Figs. 10, 11, 12, 13), it is found that after the formation of phytosomes (approximately after 100 ns), the slope of MSD curves of Eg-PC, Lu-PC, and Qu-PC systems are increased. While in the Re-PC system, where the complex of Re and PC is deformed during MD production, the MSD plot linearly as a function of simulation time is increased. Accordingly, it can be concluded the stability of the PP-PC complex in an aqueous solution can be identified from the MSD diagram.

**Figure 17:**
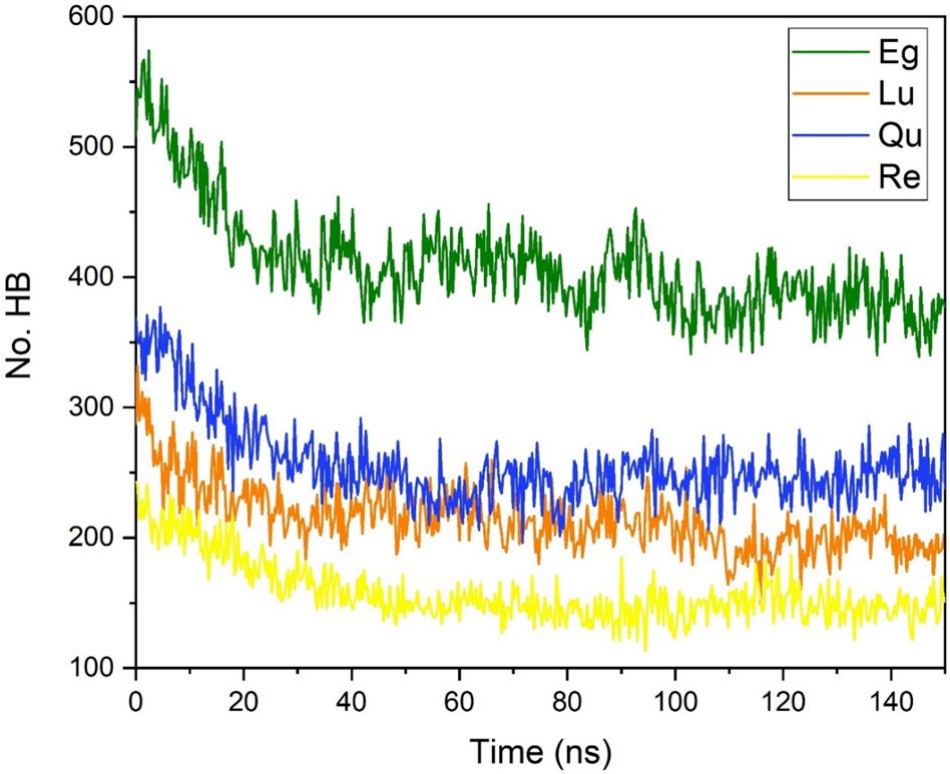
The number of hydrogen bonds of PP molecules with water molecules as a function of simulation time in the investigated systems.

The above-obtained results show that intermolecular hydrogen bonding between PP and PC is the main driving force stabilizing their complexes and aggregates. There are several functional groups on the outer surface of phytosomes that enable formation of HB with water molecules. The formation of HB between the water and PP-PC molecules is evaluated using the "gmx hbond" module of GROMCS. The cut-off = 0.35 nm and 30° are used for limiting the distance and angle of proton-donor/acceptor pairs, respectively. Changes in hydrogen bonding between PP-PC complexes and solvent molecules during simulation time are given in Fig. 17. Furthermore, in Table 2, the average, maximum (Max), and minimum (Min) of HB for PP-PC/water, PP/water, and PC/water pairs are summarized. Also, the FL parameter is reported, which is calculated using the following equation:

**Table 2:** Maximum (Max), minimum (Min), average FL values of the number of hydrogen bonds between water and different component systems.

		Min	Max	Average	FL
Eg-PC	Eg-PC	339	574	409	**1.74**
	Eg	295	509	366	1.71
	PC	24	69	43	0.96
Lu-PC	Lu-PC	149	332	216	1.18
	Lu	115	257	169	1.19
	PC	23	77	47	0.87
Qu-PC	Qu-PC	196	377	257	1.42
	Qu	153	315	211	1.30
	PC	28	74	46	1.00
Re-PC	Re-PC	113	243	158	1.22
	Re	81	179	114	1.16
	PC	26	79	44	0.83

Significant values are in bold.

7 $$FL=\frac{average \; number \; of \; HBs}{ Max-Min}$$

The lower FL value shows that the fluctuation along the time for the number of HBs is more and vice versa.

As can be seen in Fig. 17, the HB in all of the studied systems has a decreasing trend. This is expected because the PP-PC molecules at the beginning of the simulations are located in the bulk of the water. But during the MD production by forming the phytosome, the number of HB between PP-PC and water decreased. The average of hydrogen bonds has the following order: Eg-PC > Qu-PC > Lu-PC > Re-PC. This trend confirms the higher solubility of Eg-PC and Qu-PC systems in water environments. The higher solubility of Eg-PC can be related to the presence of more hydroxyl groups in the structure of the Eg molecule. Furthermore, the relatively high solubility of the Qu-PC system may arise from remaining a Qu-PC molecule in the water bulk. It should be noted that, as we expected, the number of hydrogen bonds between the drug-water pair is more than the PC-water pair (Table 2). FL values show that the Eg-PC system not only has the highest solubility but also the formed HB has the lowest fluctuation in the MD simulation time.

### Steered molecular dynamics simulations

**Figure 18:**
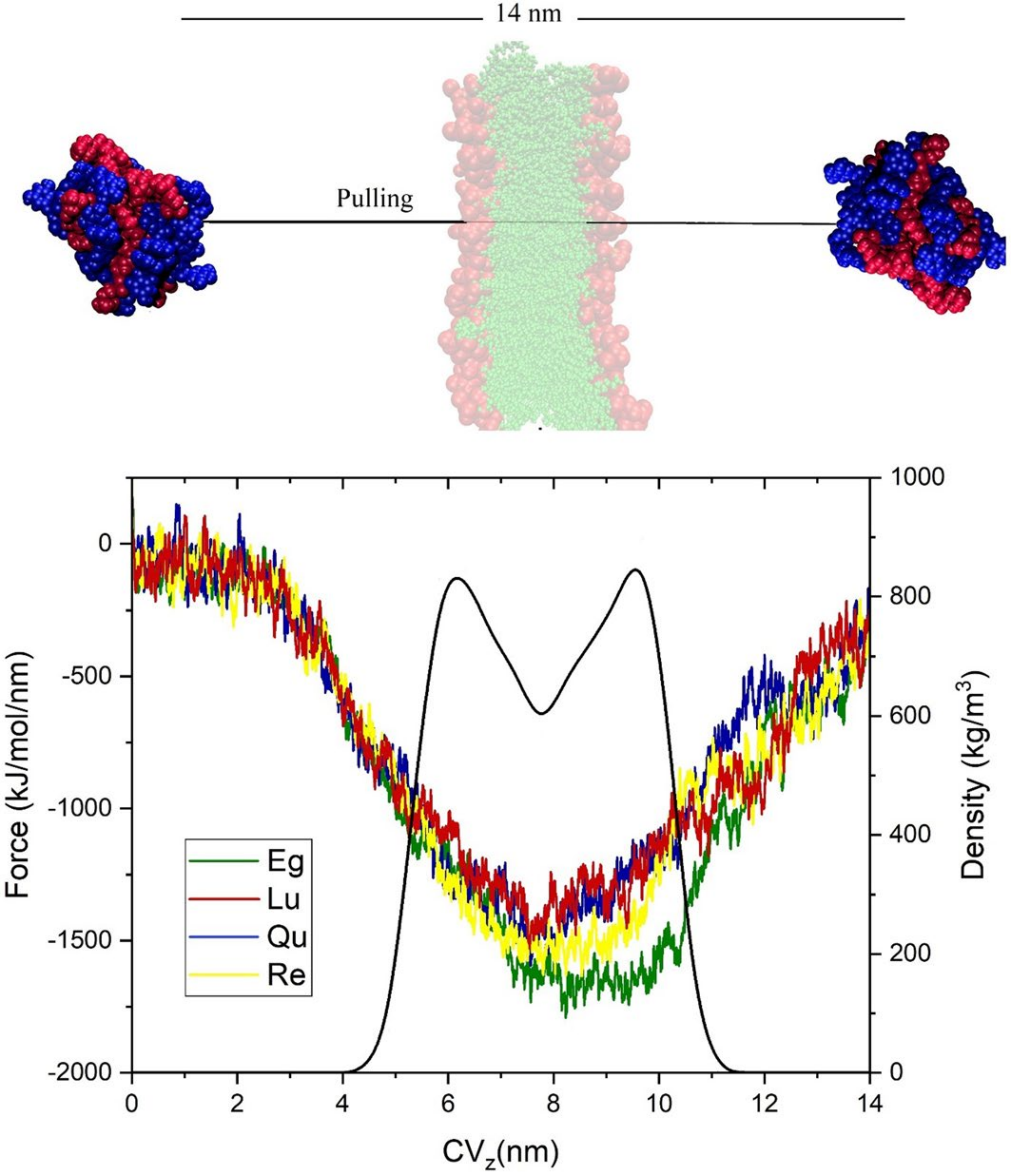
Illustration of the pulling simulation of phytosome through the cell membrane and the bottom) Force vs. collective variable (CV_z_, the COM position of the phytosome in the z-direction) during SMD. The black curve in the bottom panel represents the density profile of the membrane.

A phytosome is known as an efficient DDS when it can cross the cell membrane and localize into the cell interior to exert its effect on its target. In this context, the SMD simulation is widely employed to pull DDSs or drugs through a membrane. Using this method, it is possible to explore the ability and the dynamic behavior of a phytosome when crossing the cell membrane. To our best knowledge, this is the first series of MD simulations that examine the direct penetration of phytosomes through a membrane. The force profiles during the SMD simulations as a function of the collective variable (CV_z_, the COM position of the phytosome in the z-direction) are plotted in Fig. 18. Furthermore, the density profile of the membrane is given in this Figure.

**Figure 19:**
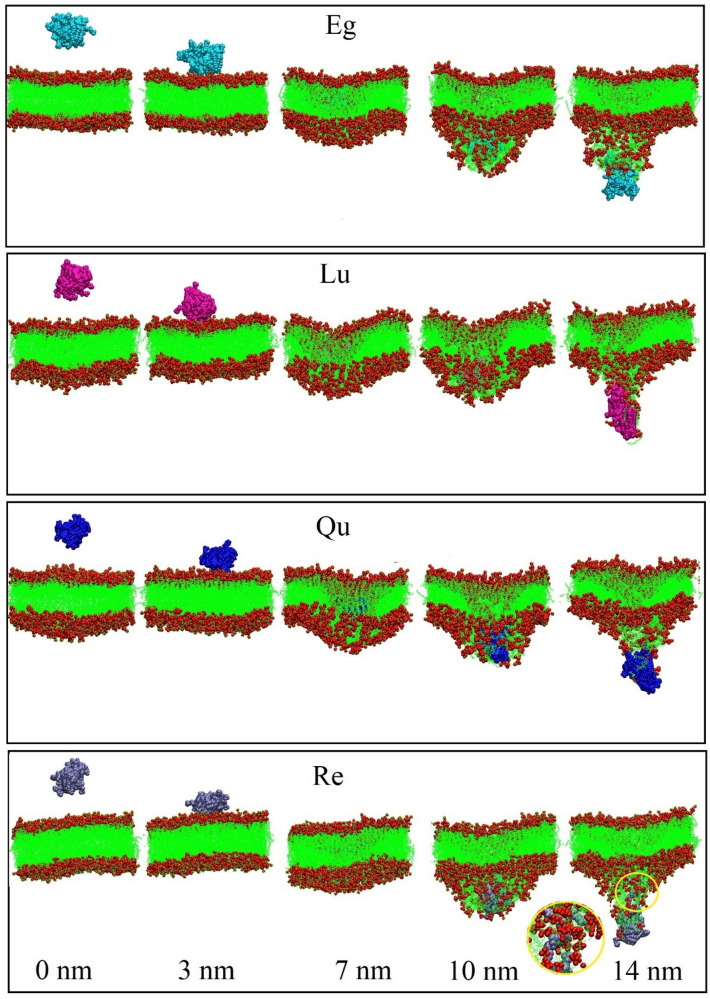
Side-view snapshots of the phytosome and membrane at different position during the SMD simulation.

As can be seen in Fig. 18, all of the studied phytosomes in the membrane-crossing process have a similar trend. During the travel of phytosomes among the bulk of water (from 0 to 3 nm), the force values fluctuate around a constant value. It can be related to dissipative force induced by its friction with the water against the phytosomes movement. From the water-bilayer interface (3–5 nm) to the center of the membrane (around 8 nm), the force profiles show a decreasing trend. This observation, in line with experimental data, confirms that the phytosome platform facilitates the penetration of PP compounds into the membrane cells. After the phytosome passes through the middle of the membrane (lipid part), the force increases. This behavior can be attributed to the hydrophobic nature of PC part of the phytosome that interacts with the lipid part of the membrane. Figure 19 shows the penetration process in which phytosomes pass through cell membranes under the influence of the applied force. It is observed that almost all of the studied systems have the same trend. In all systems, some of the membrane phospholipid groups enter the aqueous environment along with the phytosome. However, in the Eg-PC system (Fig. 19A), more phospholipids leave the membrane. This finding will be further explored via different quantity analyses. Another interesting point in this Figure is that in the Re-PC system (Fig. 19D), the phytosome is disrupted during the crossing of the membrane where some drugs are separated during this process. This behavior once again emphasizes the importance of forming strong hydrogen bonds between the components of phytosomes in increasing their biocompatibility.

**Figure 20:**
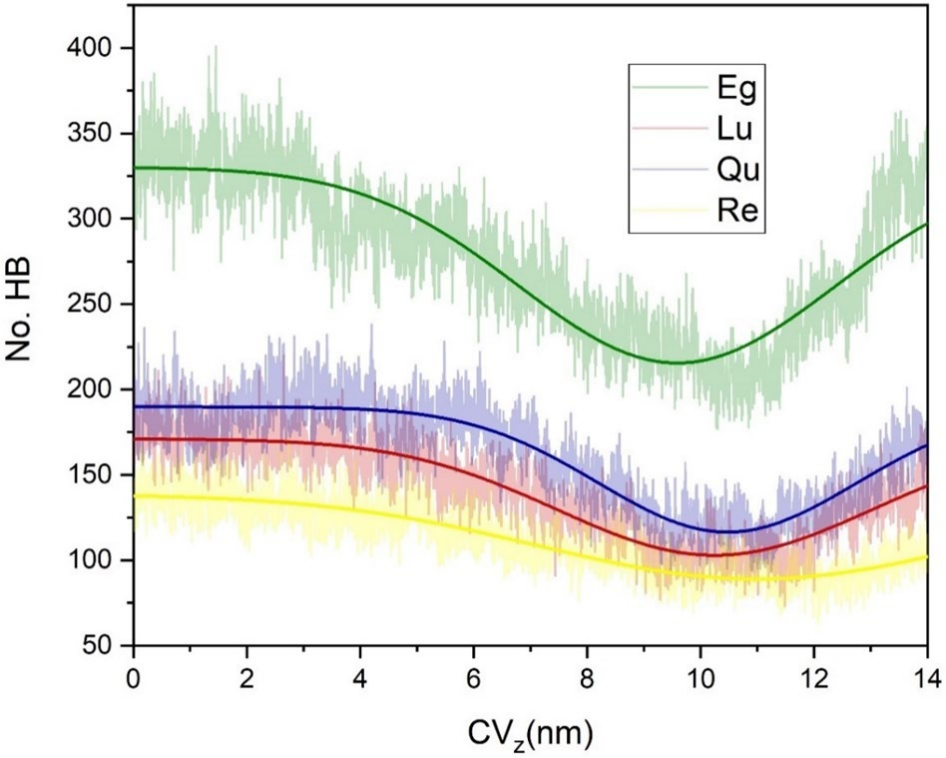
Number of HB between phytosome and the water as a function of collective variable (CVz, the COM position of the phytosome in the z-direction) during SMD.

For evaluating the role of HB in the membrane-crossing process, the number of HBs for phytosome-water is computed and given in Fig. 20. As we expected, the number HB of Eg-PC phytosome with water is more than the other systems. In all of systems, the number of hydrogen bonds with water decreases with the penetration of phytosomes into the membrane. As the phytosome passes through the membrane, the number of hydrogen bonds increases. Interestingly, it is observed that in Re-PC system, with the passage of phytosomes through the membrane, the increasing trend of hydrogen bonds is less than other systems. This finding could be related to remaining some the Re molecules inside the membrane (Fig. 19).

**Figure 21:**
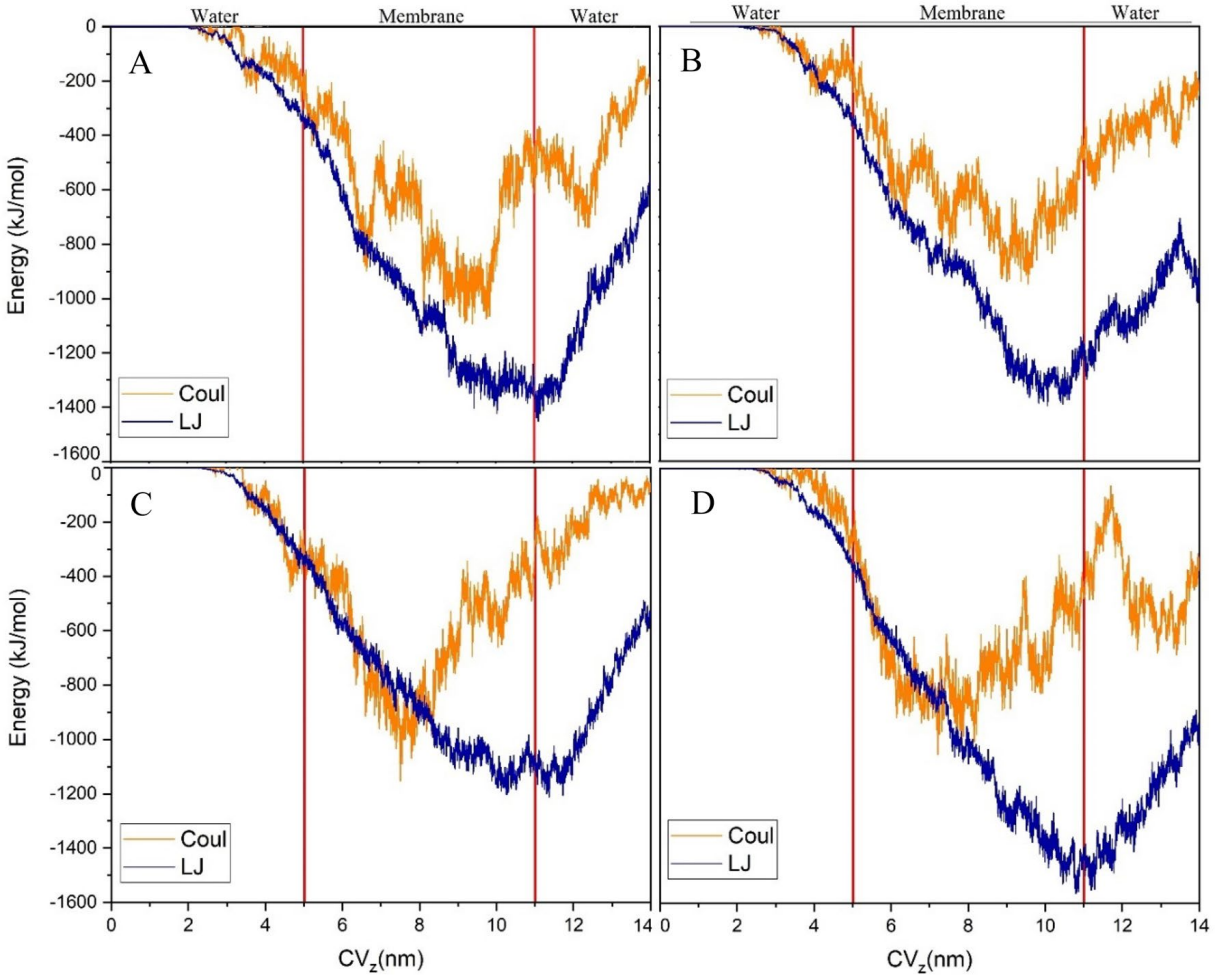
Variation of LJ and electrostatic (Coul) energies between phytosome and the membrane as a function of collective variable (CVz, the COM position of the phytosome in the z-direction) during SMD in (**A**) Eg-PC, (**B**) Lu-PC, (**C**) Qu-PC, and (**D**) Re-PC.

The variation of electrostatic and vdW energy terms for the membrane-phytosome pair as a function of the collective variable are given in Fig. 21. It is found that there is a positive correlation between the applied force and energy terms. From 0 to 2 nm, the interaction between the membrane and phytosome is zero because the phytosome is far from the membrane (outside the cut-off radius of its atoms). As phytosomes move toward the membrane and contacts between them emerge, the two energy terms begin to decrease. When the phytosome is fully placed inside the membrane, the most interaction between them is observed. It should be noted that the location of the lowest value of electrostatic energy on the CV line, which arises from the interaction of phytosome with head groups of the lipid bilayer, is located at closer distances to the vdW energy. After the phytosome passes through the membrane, the energy between them rises, along with the increase in force. In all of the systems, it is observed that the energy values between the membrane and the phytosome are a significant amount that confirms some of the membrane components along with the DDS entering to the cell. In Re-PC system, due to disrupting the phytosome, the energy values are higher than the other systems.

## Conclusions

A computational study is performed to explore the nature of intermolecular interactions in the formation of phytosomes between PC and a series of polyphenols, including Eg, Lu, Qu, and Re. Furthermore, the dynamic behavior of the formed phytosomes in the biological environment and crossing the membrane cell is investigated. The DFT results indicate intermolecular HBs of the polyphenol compounds with phosphate and glycerol parts of PC are the main driving force in the formation of phytosomes. These interactions are reinforced by the vdW interaction between the choline part and drugs. The strongest interactions are formed in the Eg-PC system, where the flexible structure of Eg makes more and stronger intermolecular interaction possible. All-atoms MD simulations are performed to capture the self-aggregation of phytosome in an aqueous solution. The obtained results reveal that the PP-PC complexes in all of the systems tend to self-assemble and formed stable phytosome systems. It is found that in Eg-PC, Lu-PC, and Qu-PC systems, due to the formation of strong hydrogen bonds (HBCP < 0 and ∇2ρBCP > 0) between PP and PC, the PP-PC complexes protect from degradation in the aqueous environment. While the Re-PC complex is unstable and degraded in the presence of water molecules. It is observed that the hydrophobic tails of PC form the core of the phytosome and are wrapped by drugs and hydrophilic parts. The higher stability and solubility belong to Eg-PC systems which can be related to the strong intermolecular interactions and presence of more hydroxyl groups in the structure of the Eg molecule. The SMD simulation results are in line with experimental data and show that the phytosome platform facilitates the penetration of PP compounds into the membrane cells.

## Supplementary Information

Supplementary Information.

## Data Availability

All of Computational models are available from the corresponding author upon request.
