# Supplementary material for: The combination of polyphenols and phospholipids as an efficient platform for delivery of natural products
Source: Sci Rep. 2023 Feb 13;13:2501. doi: 10.1038/s41598-023-29237-0 (PMC9925764; doi:10.1038/s41598-023-29237-0)
Supplement: Supplementary file 1 — Supplementary Information. [file 41598_2023_29237_MOESM1_ESM.docx]

**Supplementary material**

The Combination of Polyphenols and Phospholipids as an Efficient Platform for Delivery of Natural Products

*Hassan Hashemzadeh^1^,* [*Mohammad Yahya Hanafi-Bojd*](https://pubmed.ncbi.nlm.nih.gov/?term=Hanafi-Bojd+MY&cauthor_id=35093324)^2,3,^^[[1]](#footnote-1)^*, Milad Iranshahy^4^, Asghar Zarban^5^ Heidar Raissi^6^*

^1^Department of Pharmaceutics and Pharmaceutical nanotechnology, School of Pharmacy, Birjand University of Medical Sciences, Birjand, Iran, Email: [hashemzade_h@bums.ac.ir](mailto:hashemzade_h@bums.ac.ir)

^2^Cellular and Molecular Research Center, Department of Molecular Medicine, Birjand University of Medical Sciences, Birjand, Iran, Email: [my_hanafi_bojd@yahoo.com](mailto:my_hanafi_bojd@yahoo.com)

^3^Department of Pharmaceutics and Pharmaceutical nanotechnology, School of Pharmacy, Birjand University of Medical Sciences

^4^ Department of Pharmacognosy, School of Pharmacy, Mashhad University of Medical Sciences, Mashhad, Iran, Email: [iranshahiml@mums.ac.ir](mailto:iranshahiml@mums.ac.ir)

^5^Department of Clinical Biochemistry, Faculty of Medicine, Birjand University of Medical Sciences, Birjand, Iran, Email: [azarban@bums.ac.ir](mailto:azarban@bums.ac.ir)

^6^Department of Chemistry, University of Birjand, Birjand, Iran, Email: [hraeisi@birjand.ac.ir](mailto:hraeisi@birjand.ac.ir)

**Table S1.** Details of the MD simulation systems.

| System | dimension box  size (nm^3^) | Number of PP-PC complex | Polyphenol | Number of Water | Na^+^ + Cl^-^ ions |
| --- | --- | --- | --- | --- | --- |
| Eg-PC | 10×10×10 | 10 | Eg | 31935 | 90+90 |
| Lu-PC | 10×10×10 | 10 | Lu | 32020 | 90+90 |
| Qu-PC | 10×10×10 | 10 | Qu | 32029 | 90+90 |
| Re-PC | 10×10×10 | 10 | Re | 32048 | 90+90 |


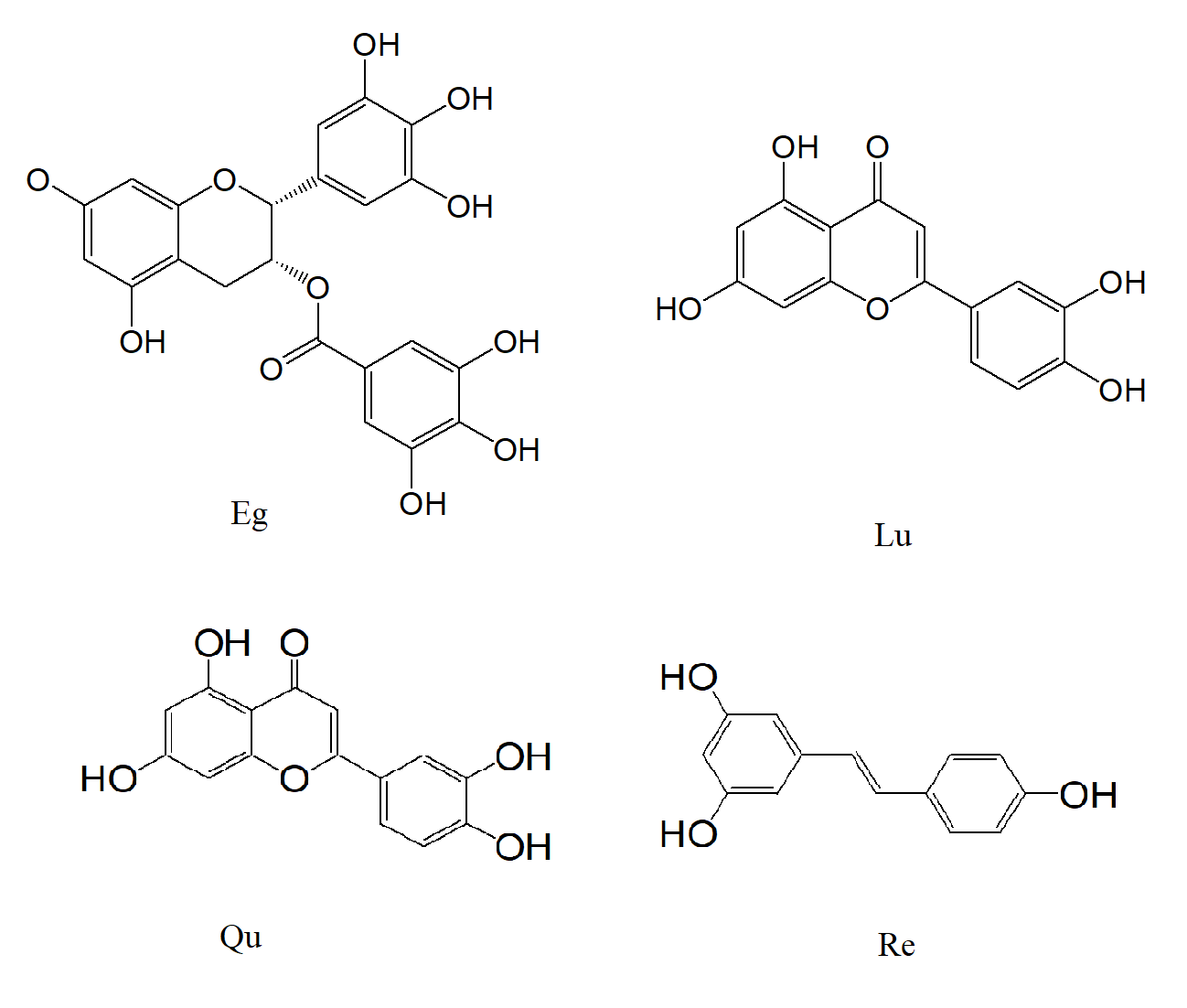


**Figure S1**. 2D structures of the studied polyphenols.


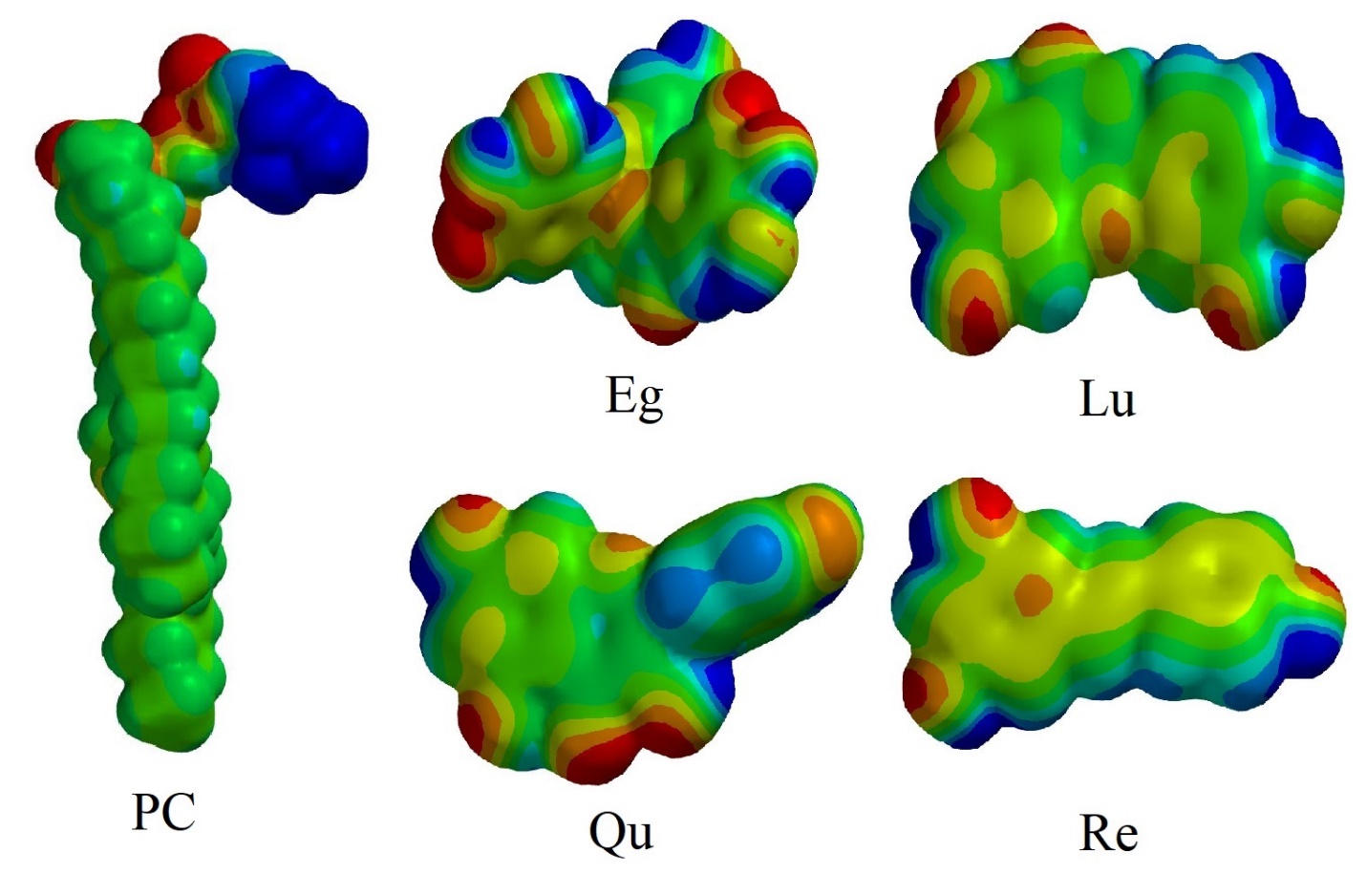


**Figure S2.** Map of the electrostatic potential (MEP) for the studied molecules.


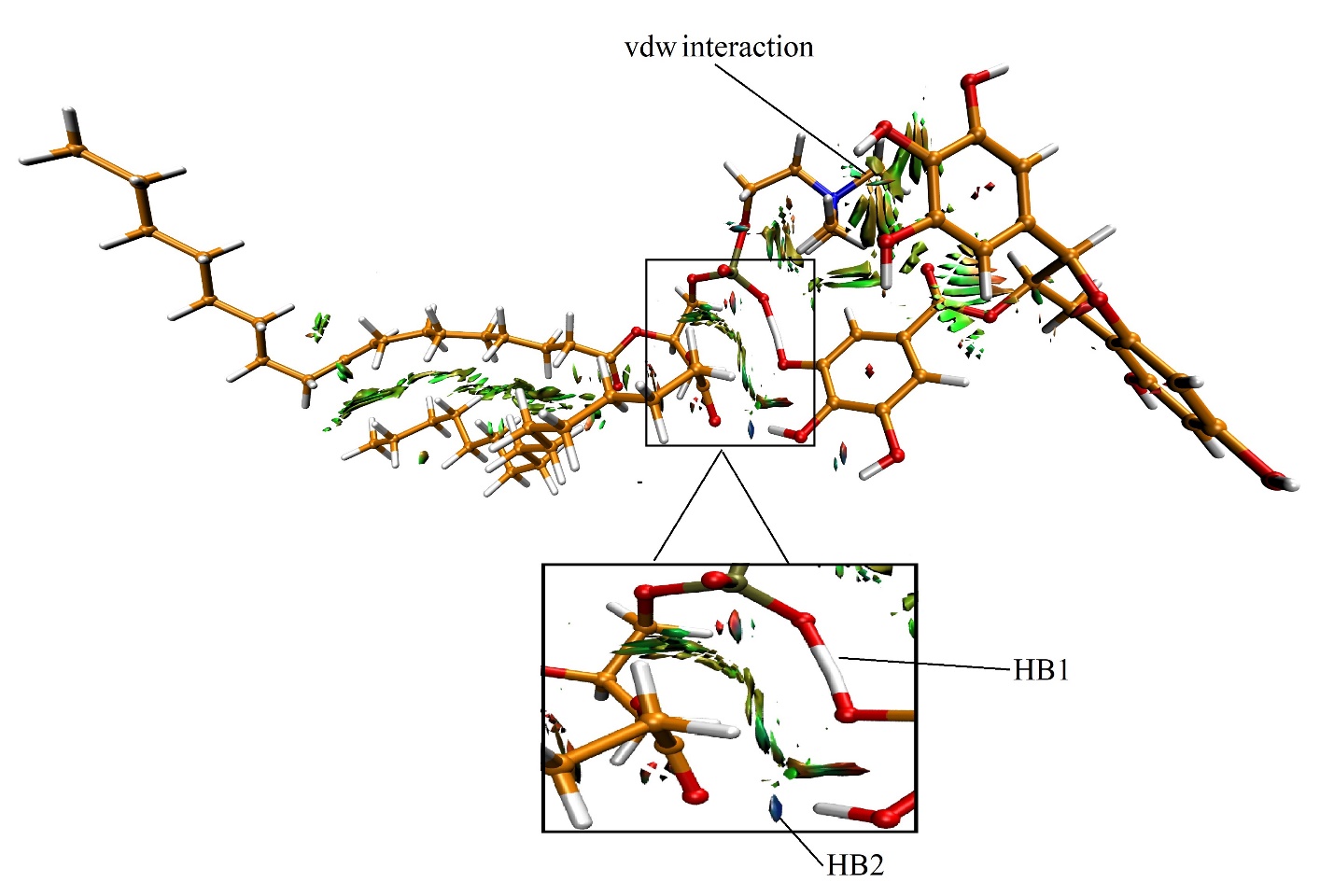


**Figure S3.** Color-filled RDG iso-surfaces of Eg-PC complex. Green regions refer to van der Waal interactions. The steric interactions are shown in red and blue region refer to the strong attractive interactions.


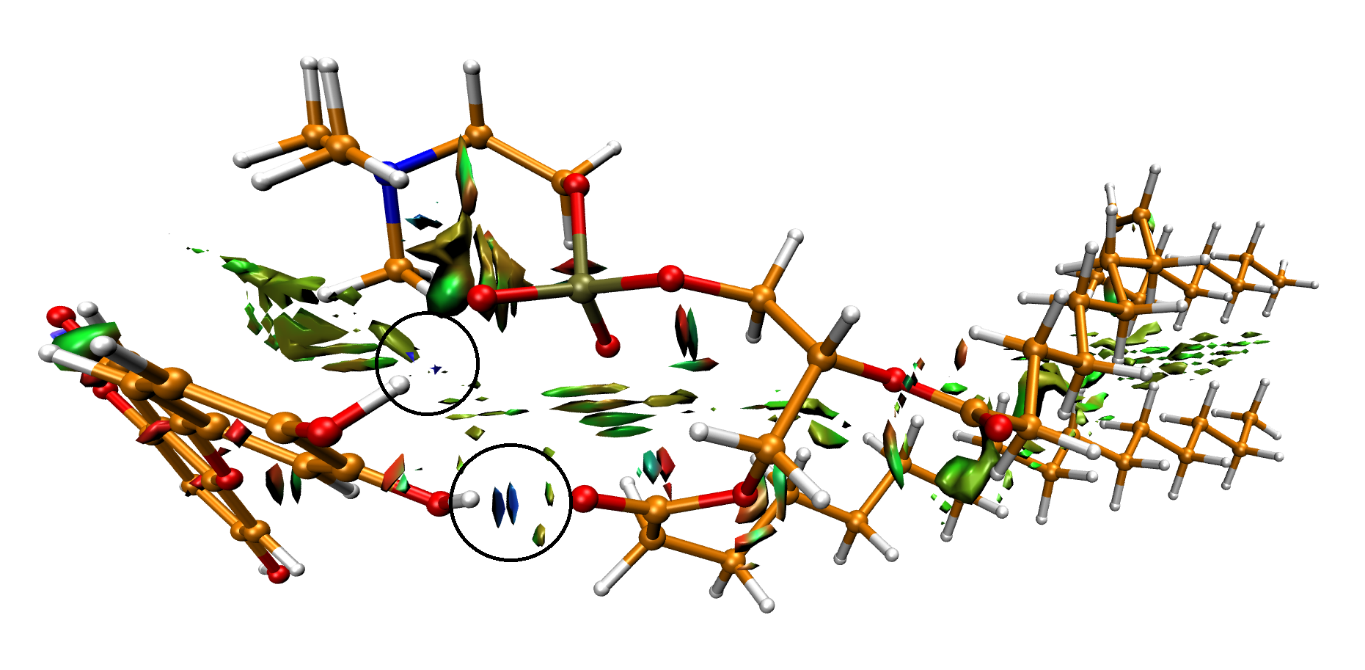


**Figure S4.** .Color-filled RDG iso-surfaces of Lu-PC complex. Green regions refer to van der Waal interactions. The steric interactions are shown in red and blue region refer to the strong attractive interactions.


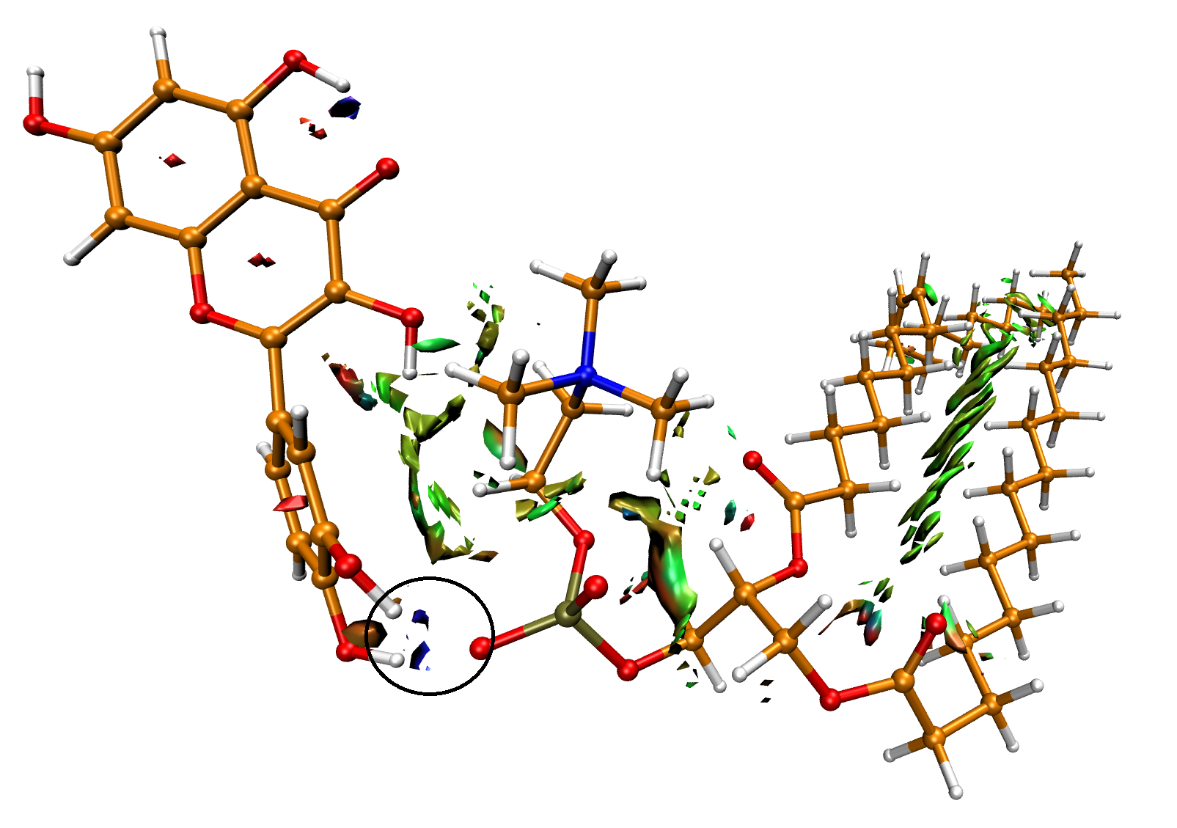


**Figure S5**. Color-filled RDG iso-surfaces of Qu-PC complex. Green regions refer to van der Waal interactions. The steric interactions are shown in red and blue region refer to the strong attractive interactions.


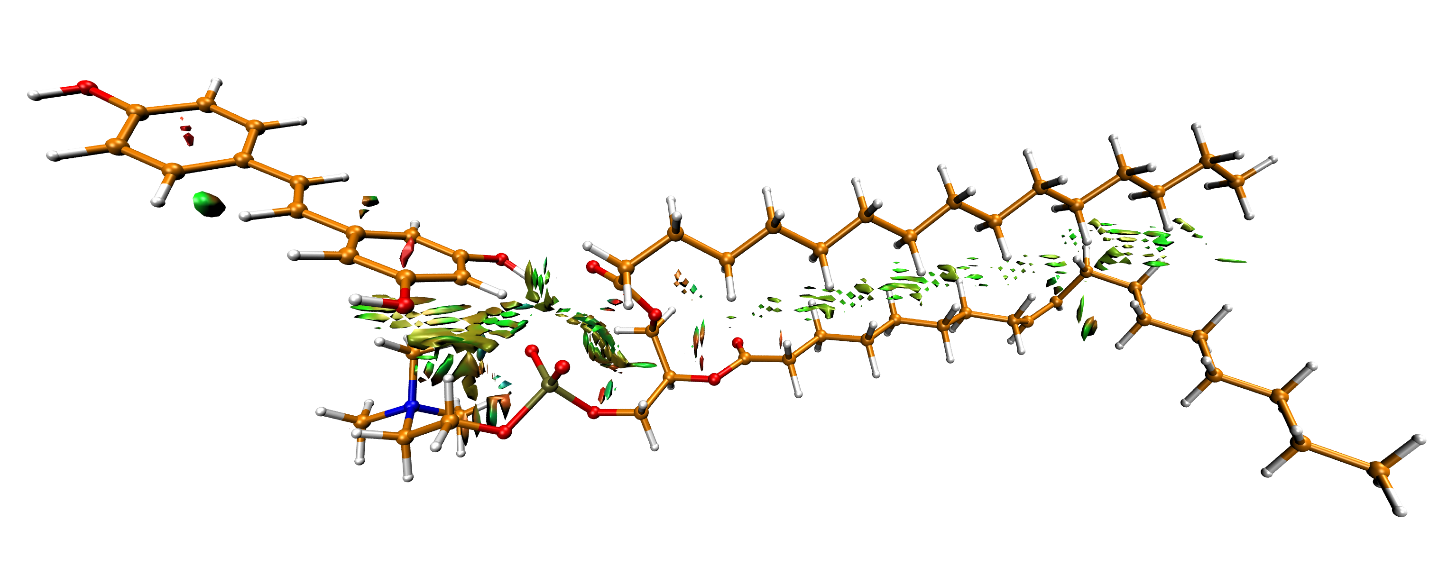


**Figure S6**. Color-filled RDG iso-surfaces of Re-PC complex. Green regions refer to van der Waal interactions. The steric interactions are shown in red and blue region refer to the strong attractive interactions.


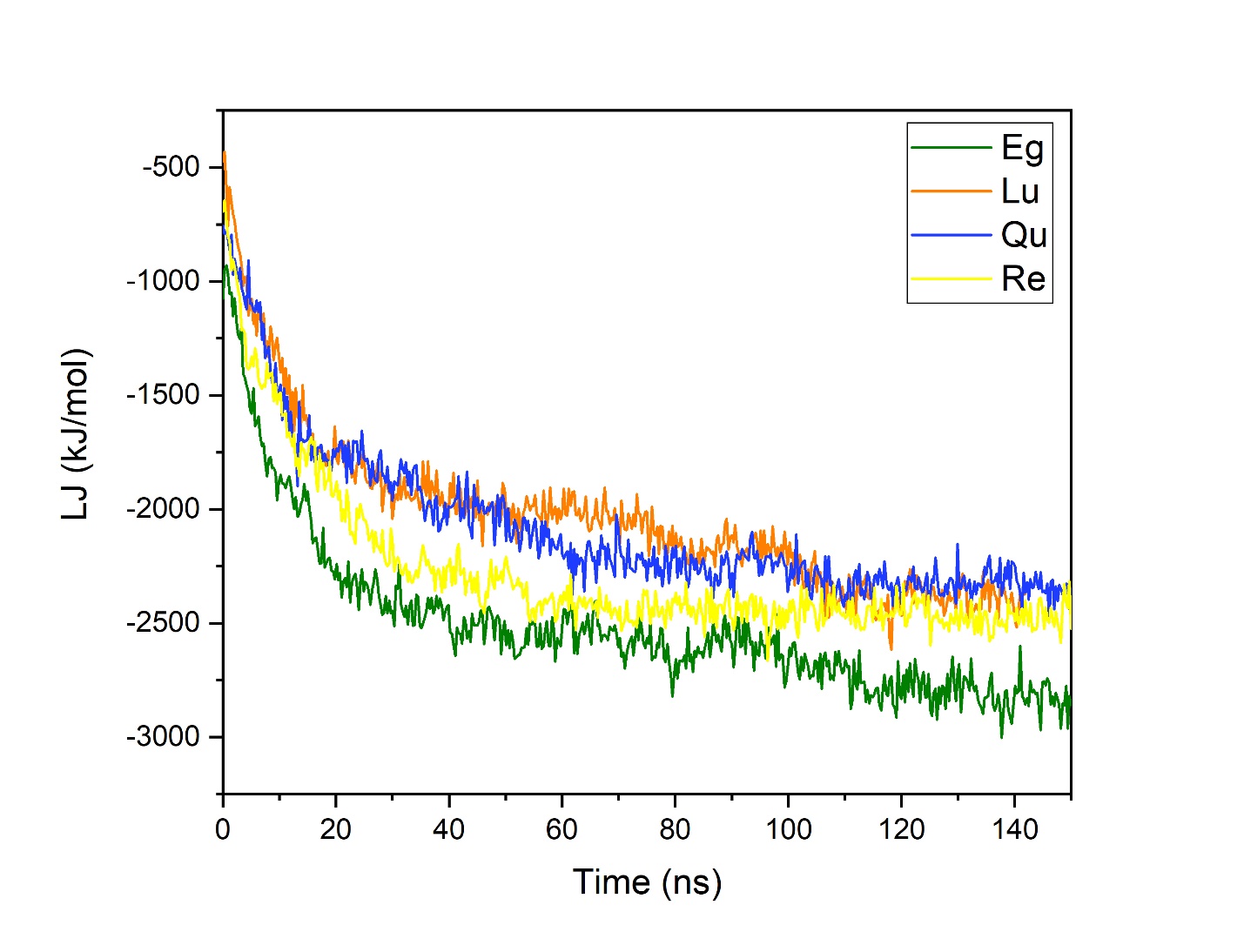


**Figure S7.** VdW energy between PP-PC and PP-PC molecules vs. simulation time

1. Corresponding Author: [my_hanafi_bojd@yahoo.com](mailto:my_hanafi_bojd@yahoo.com) [↑](#footnote-ref-1)
